# An empirical study on the evaluation and influencing factors of digital competence of Chinese teachers for TVET

**DOI:** 10.1371/journal.pone.0310187

**Published:** 2024-09-13

**Authors:** Yu Xin, Yuanbin Tang, Xiangwei Mou

**Affiliations:** 1 The College of Mechatronics and Automobile Engineering, Liuzhou City Vocational College of, Liuzhou, Guangxi, China; 2 The Teachers College for Vocational and Technical Education, Guangxi Normal University of, Guilin, Guangxi, China; RMIT University, VIET NAM

## Abstract

The digital competence of TVET (technical and vocational education and training) teacher is the key to the digital development of TVET. Improving the digital competence of TVET teachers is of great significance to accelerate the digital transformation process of TVET. However, there are still few researches on digital competence of TVET. The aim of this study is to describe the self-cognition of digital competence of Chinese teachers for TVET. For this purpose, based on the development characteristics of China’s TVET, a standard system for digital competence of TVET teachers in China was established, including digital mindset and attitude, digital knowledge and skills, digital education and teaching, digital care and support, and digital collaboration and development. Based on this, an assessment tool was developed to investigate the current situation of competence of 2,514 in-service TVET teachers in China. The results show that the competency system is an effective tool to measure the digital competence of Chinese TVET teachers, and other internal and external factors except gender have different degrees of influence on the digital competence of teachers. According to the results of this study, different suggestions are put forward to train the digital competence of TVET teachers.

## 1.Introduction

With the development and application of new-generation digital technologies such as cloud computing, the Internet of Things (IoT), and big data, the transformation and innovation of various industries driven by digitalization have become a global theme. Education is recognized as a key driver of development in the digital age. Technical and vocational education and training (TVET) is the provision of training and education for individuals to acquire the skills and knowledge needed for a particular occupation or industry, and is an important source of technical and skilled personnel training in the digital economy, with irreplaceable benefits in supporting economic and social development. The digital competence of TVET teachers concerns both the professional development of individual teachers and the enhancement of the competence of skilled personnel, and directly affects the degree of development of digitalization of TVET, as well as the excellent competitiveness of TVET in the era of the digital economy. In 2021, The Office of Educational Technology of the U.S. Department of Education released the Teachers’ Digital Learning Guide [1] and the School Leader Digital Learning Guide [2]. These two documents aim to systematically expand educators’ teaching horizons and enhance their teaching and management capabilities in digital education environments by building a system of rich educational resources and a set of practical tools.In recent years, France has been continuously promoting reforms to further enhance the attractiveness of TVET. France has continued to promote reform, following the release of 11 reform documents in 2018, in May 2023, it released the "12 Measures to Make Vocational High Schools the Future Choice of Young People and Enterprises", which emphasizes learner centeredness, enhances teacher education momentum, and advocates for stakeholders to jointly build vocational schools to enhance the attractiveness of TVET [3]. The UK Data Strategy released by the UK government in December 2020 marks the beginning of a focused breakthrough in the direction of improving the digital level of TVET, and strengthening the digital skills of workers to enhance the adaptability of TVET, based on the digital transformation of the economy and society [4].

With the process of digital transformation in education and the emergence of conceptual frameworks for digital competence at international and national levels, many countries or organizations have explicitly pointed out that teachers’ digital competence enhancement is the key to the digital transformation of education, and that teachers should have the appropriate digital competence to ensure the integration of digital technologies into teaching and learning, which will in turn enhance the digital competence of students. UNESCO updated the ICT Competency Framework for Teachers (ICT-CFT-3 Framework) in 2018 [5], which is a guiding tool for the training of new and post-service teachers and includes six areas: understanding ICT in education, curriculum and assessment, pedagogy, application of digital skills, organization and management, and teacher professional learning. As well as three progressive levels of pedagogical use and technology mastery: knowledge acquisition, knowledge deepening and knowledge creation. Similarly, there are digital competence frameworks from the European Union (DigCompEdu) [6], among others. In addition to digital competency frameworks for teachers at the international level, digital competency frameworks from academic research are also emerging. The TPACK model proposed by Mishra and Koehler describes a model of teacher knowledge for integrating technology into teaching and learning [7], and the TPACK framework of teacher knowledge is described in detail as a complex interplay between three bodies of knowledge: content, pedagogy, and technology. Janssen et al [8]. developed a digital competency model from an expert’s perspective, which includes 12 “core” and “supportive” competencies to support citizens’ screening and seamless use of digital technologies.Existing research has shown that the rapid development of digital technology has an unprecedented impact on the world of work, leading to a shift in the complexity of workers’ skills [9]. This implies that TVET, as the foundation for training skilled talents, must continually integrate digital technology deeply into education methods and content to provide better vocational skills training for workers, enhancing their employability and adaptability to their jobs. Additionally, this approach can provide businesses with more and better technical talent, facilitating industrial upgrading and transformation. In contrast to regular teachers, TVET teachers need not only to master the ability to use digital technology and resources for remote teaching but also need to possess digital literacy and digital competence relevant to different professional fields and workplaces. This involves integrating digital tools and technology into TVET and allowing learners to interact with advanced tools and equipment used in real workplaces, practicing complex tasks in a controlled environment, and continuously enhancing learners’ vocational skills.

China has currently established the world’s largest TVET system, and many policy documents mention that accelerating the digitization of TVET is a necessary path to building high-quality TVET [10]. In December 2022, the Chinese Ministry of Education released the industry standard "Teacher Digital Literacy" [11], which clarifies the digital literacy of general education teachers, that is, the awareness, ability, and responsibility of teachers to appropriately use digital technology to acquire, process, use, manage, and evaluate digital information and resources, discover, analyze, and solve educational and teaching problems, optimize, innovate, and transform educational and teaching activities. Compared with ordinary teachers, TVET teachers not only need to master the ability to use digital technology and resources to carry out remote teaching, but also need to master the digital literacy and digital abilities required for different professional fields and workplaces. TVET teachers face more diverse teaching scenarios and diverse ability requirements for their digital abilities.

Driven by global education reforms and national policies and projects, China has made significant progress in the digital development of TVET. The "Report on the Development of Informationization in TVET (2021 Edition)" shows that TVET teachers in China have made significant progress in digital teaching, digital resource development and management, among other aspects. For instance, 74.54% of TVET teachers frequently use multimedia devices such as computers, projectors, and electronic whiteboards for teaching. Over half of the teachers regularly use course resources from online teaching platforms. Nearly 80% of teachers use the internet for professional learning and educational research [12]. However, challenges remain in the digital competence of TVET teachers in China, including uneven development of teacher capabilities and regional disparities in teacher competence levels [13]. This issue fundamentally stems from the lack of standardized digital competence assessment criteria and methods tailored to TVET teachers, resulting in a lack of objectivity and fairness in teacher competence assessment mechanisms. This challenge is a common issue inhibiting the development of TVET teachers’ digital competence worldwide [14]. Compared with European countries, China’s TVET are based on schools, emphasizing the imparting of vocational knowledge and skills, while the apprenticeship system in European countries extends TVET venues between enterprises and schools. This means that the digital competency framework of Chinese TVET teachers should be more comprehensive to meet various teaching needs.

Therefore, this study aims to clarify the basic dimensions of TVET teachers’ digital competence based on the characteristics of Chinese TVET teachers. It seeks to develop a targeted digital competence assessment scale for TVET teachers, understand the current status and influencing factors of TVET teachers’ digital competence in China, and aim to enhance the digital abilities of Chinese TVET teachers for the digital age. This research also intends to provide a model and experience for the development of TVET teachers digital competence levels globally, promote cooperation and exchange in the field of TVET between China and other countries, and collectively achieve sustainable education development goals.

## 2.Literature review

### 2.1 Digital competence

In 2006, the European Union officially introduced the term "digital competence" in its report on "Key Competences for Lifelong Learning." Digital competence was categorized as one of the eight core competences and defined as "the confident and critical use of information communication technologies for self-fulfillment and professional practice." European policies began to emphasize not only the ability to acquire and use technology but also the ability to apply technology to benefit one’s life, work, and learning. It was referred to as a fundamental life skill in the digital age [15]. In the EU’s analysis of digital competence, it was noted that digital tools are becoming daily intermediaries for all types of tasks and are integral to any set of skills. Calvani et al. defined digital competence as the ability to adapt flexibly to new technological environments, analyze, select, and critically evaluate data and information, solve corresponding problems, and develop awareness of personal responsibility and respect while building and sharing collaborative knowledge [16]. In 2012, the EU Digital Competence Research Team proposed that digital competence involves knowledge, skills, and attitudes (including abilities, strategies, values, and awareness) for using information technology and digital media to perform tasks, solve problems, communicate, manage information, collaborate, create and share content, and construct knowledge [17].

In China, digital competence was initially translated as "digital literacy" and considered an extension of the concept of information literacy. It was viewed as a comprehensive, dynamic, and open concept encompassing cognitive skills, emotional skills, social skills, and even applications in pedagogical theory [18]. In 2017, Ren Youqun first introduced the term "Digital Competence" in the context of education, asserting that it should be considered one of the core competences that make up a student, including the ability to learn, creativity, interdisciplinary problem discovery, and problem-solving abilities [19]. In 2021, the Central Cyberspace Affairs Commission of China issued the "Action Plan to Enhance the Digital Literacy and Skills of the Whole Population," which used the concept of "digital literacy and skills" and defined it as "a collection of qualities and abilities that individuals should possess in learning, working, and living in a digital society, including digital access, creation, use, evaluation, interaction, sharing, innovation, security, ethics, and other aspects" [20]. Zheng Xudong pointed out that digital competence is not a basic or general ability but rather a relatively advanced comprehensive ability [21].

In general, research in China emphasizes that digital competence is a more comprehensive, multidimensional, learner-centered digital ability. While some Chinese scholars still prefer to use the translation "digital literacy," the academic community tends to regard digital competence as a complex combination of abilities and literacy related to digital technology. This competence is considered advanced, evolving with the development of the digital age, and increasingly directed toward competitive skill sets. Therefore, this study chooses to use the internationally accepted term "competence" rather than "literacy."

### 2.2 Research on teacher digital competence framework

In 2017, the European Union developed and published the European Framework for Digital Competence of Educators, dividing teacher digital competence into six domains: professional engagement, digital resources, teaching and learning, assessment, empowering learners, and promoting learners’ digital competence [22]. In the same year, the International Society for Technology in Education (ISTE) updated the "National Educational Technology Standards for Educators" [23], categorizing teacher roles into Empowered Professional and Learning Catalyst. Teachers were expected to take on seven roles: learner, leader, citizen, collaborator, designer, facilitator, and analyst. Among these, learner, leader, and citizen roles emphasized technology’s empowerment for professional development. Collaborator, designer, facilitator, and analyst roles highlighted teachers’ capacity to promote student development. In Norway, the Center for ICT in Education published the "Teacher Professional Digital Competence Framework" in the same year, dividing teacher digital competence into seven dimensions: subject and basic skills, school in the social context, ethics, pedagogy and subject pedagogy, leadership in the learning process, interaction and communication, and change and development [24]. In addition, countries such as the UK in 2019 and Spain in 2017 also released specific frameworks for teacher digital competence [25,26]. Some scholars have further refined and expanded influential international digital competence frameworks using empirical methods. For example, Katia Verónica Pozos Pérez analyzed the elements of digital competence for higher education teachers based on teaching competence [27]. Other studies focused on exploring the structure of teacher digital competence in specific contexts, such as tourism education [28], and pre-service teachers, including Norwegian English intern teachers’ digital competence, covering basic digital skills, didactic ICT competence, learning strategies, and digital education [29]. Elen Instefjord integrated the TPACK framework and Rune J. Krumsvik’s teacher digital competence framework to develop a framework for preservice teacher digital competence, including technology proficiency, pedagogical compatibility, and social awareness [30]. Zheng Xudong identified the primary components of teacher digital competence for primary and secondary school teachers in China, including digital technology capability, digital teaching capability, digital learning and innovation, digital values and pursuits, and basic personality traits. He further divided these components into 25 secondary components, constructing a model of digital competence for primary and secondary school teachers in China [31]. Scholars like Sun Xiaohong constructed teacher-centered digital competence frameworks based on international teacher digital competence frameworks, integrating teacher digital knowledge, digital skills, and digital attitudes, focusing on the three aspects of teachers’ digital cognitive competence, digital functional competence, and digital social competence [32].

In China, to accelerate the digital transformation of TVET, the government issued the "Guidelines for the Construction of Digital Campuses in Vocational Colleges" in 2020, specifying requirements for teacher digital development in five areas: information awareness and attitude, information knowledge and skills, information application and innovation, information research and development, and information social responsibility [33]. However, this document mainly focuses on teachers’ information technology capabilities based on a "teacher-centered" approach, without adequately highlighting teachers’ concern for student learning. In 2022, the Chinese Ministry of Education released the "Industry Standard for Teacher Digital Literacy," providing a framework for teacher digital literacy and defining requirements across five dimensions: digital awareness, digital technology knowledge and skills, digital application, digital social responsibility, and professional development. This document emphasizes teachers’ awareness, capabilities, and sense of responsibility in optimizing, innovating, and transforming teaching activities using digital technology [34]. However, due to its universal value orientation, the standards lack the specificity required for TVET.

**Fig 1:**
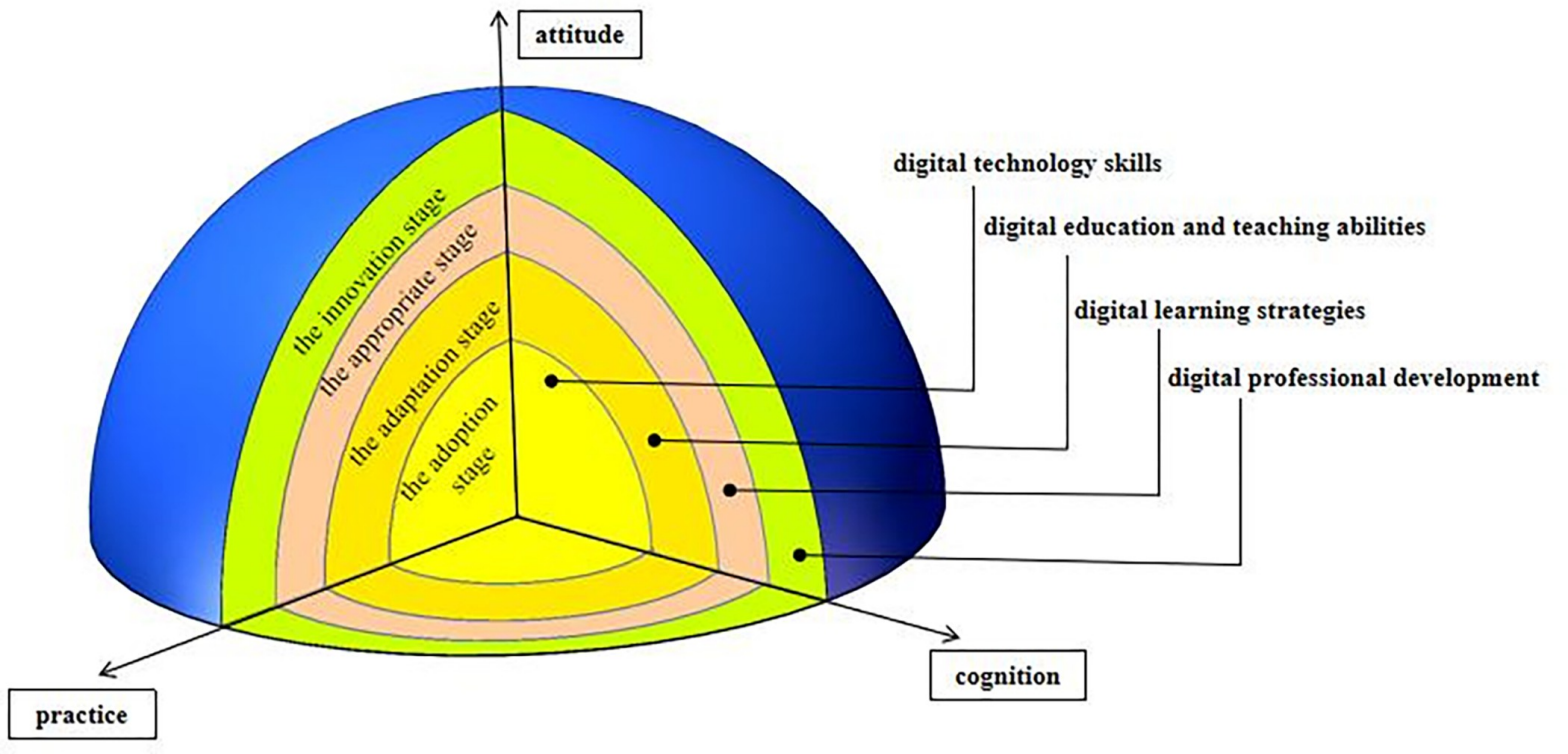
Theoretical model of digital competency framework for Chinese TVET teachers.

Krumsvik, R. J. constructed a theoretical model of teacher digital competence under the guidance of distributed theory. The model includes two axes and three domains, with self-awareness (vertical axis) and proficiency (horizontal axis) as the two axes, and digital skills, digital teaching ability, and learning strategies as the three domains. The model explains the main content of current digital competence frameworks at home and abroad. In the new era of rapid development and application of modern information technologies such as artificial intelligence, big data, and cloud computing in the future, TVET teachers are required to continuously adjust course structure, teaching content, and teaching modes according to professional talent training standards; Through enterprise rotation training, overseas training visits, industry integration, continuous improvement of professional skills, technical breakthroughs, and application technology promotion, strengthening teachers’ lifelong learning ability and professional practicality. Based on this, it is necessary to reconstruct the theoretical framework of digital competence for TVET teachers in combination with the professional standards of TVET teachers. This study adopts the main architecture of the teacher digital competence theory model under the guidance of distributed cognition theory, and based on the three major competency domains of digital application, promoting student development, and promoting teacher development that are commonly concerned by relevant standards. Guided by the concept of digital competence for TVET teachers, it is believed that the theoretical framework of digital competence for TVET teachers should include four competency domains: digital technology skills, digital education and teaching abilities, digital learning strategies, and digital professional development, emphasizing the main content of different stages of TVET teacher professional development (Fig 1).

The four dimensions of teacher digital technology skills, digital education and teaching abilities, digital learning strategies, and digital professional development abilities in the theoretical framework will develop with the deepening of attitude, cognition, and practice (three axes). In the framework, mastering digital technology skills corresponds to the adoption stage, mastering digital education and teaching abilities corresponds to the adaptation stage, mastering digital learning strategy related abilities corresponds to the appropriate stage, and mastering digital professional development related abilities corresponds to the innovation stage. The different stages of ability development reflect the transformation of vocational education teachers from focusing on the tool functions of digital technology to integrating innovative functions.

### 2.3 Factors influencing teacher digital competence: A review of related research

Some studies have focused on investigating the factors that influence teacher digital competence, categorizing these factors primarily into personal and background factors. Personal factors encompass gender, age, and more, while background factors encompass aspects like the school’s digital infrastructure, Technical training received by teachers. Various research findings indicate significant gender-based disparities in digital competence, with a tendency towards male teachers having higher capabilities [35,36]. However, there are also studies that report the opposite trend [37]. Regarding age, most studies suggest that younger teachers exhibit higher levels of digital competence compared to their older counterparts [38,39]. Some studies also indicate that teachers’ acceptance and use of technology are rooted in their attitudes towards use [40], and their levels of cognition [41], efficacy [42], and other factors also affect their acceptance and use of digital competence [43]. Therefore, some studies suggest that cultivating teachers’ motivation to use digital technology enables them to maintain a long-term [44] and efficient positive attitude towards the use of technology, ultimately achieving experiential growth in their abilities [45]. Concerning background factors, most studies suggest a positive correlation between digital infrastructure and teachers’ digital competence [46]. This is because digital infrastructure is the prerequisite and foundation for teachers to use information technology to carry out education and teaching, and is an important external guarantee provided by universities for teachers’ teaching [47]. Research has found that universities in economically underdeveloped areas face many limitations in their information technology construction, which to some extent restricts the improvement of teachers’ digital competence level [48]. In addition, there are studies that focus on the level of support for teachers’ use of digital technology, especially the teaching and training related to digital technology that teachers receive in pre-service and post service training [49], which has the potential to have a positive impact on cultivating students’ digital competence [50]. In addition, the attitude of school leaders towards digital technology may greatly affect the frequency and level of teachers’ use of digital technology, that is, the higher the availability of information technology in the classroom and school management, the higher the level of teachers’ digital competence [51]. However, current research on teachers’ digital competence mainly focuses on individual factors, and there is relatively little research on external factors, especially those related to schools. Analyzing the factors that affect teachers’ digital competence and gaining inspiration from them is the core goal of this type of research.

In summary, the aforementioned studies lay a solid foundation for the present research. However, most of these studies do not explicitly emphasize teacher types and they do not adequately consider the unique characteristics of the competence structure of TVET teachers (specific digital knowledge and skills related to their work domains). This oversight neglects the developmental characteristics of their digital competence. Therefore, this study, with the goal of developing an assessment scale for digital competence tailored to TVET teachers, raises the following research questions:

RQ1: What is an appropriate assessment scale for digital competence that aligns with the characteristics of TVET teachers?
RQ2: What is the current status of digital competence among TVET teachers in China?
RQ3: What factors influence the digital competence of TVET teachers in China?

## 3.Methodology

### 3.1 Participants

**Fig 2:**
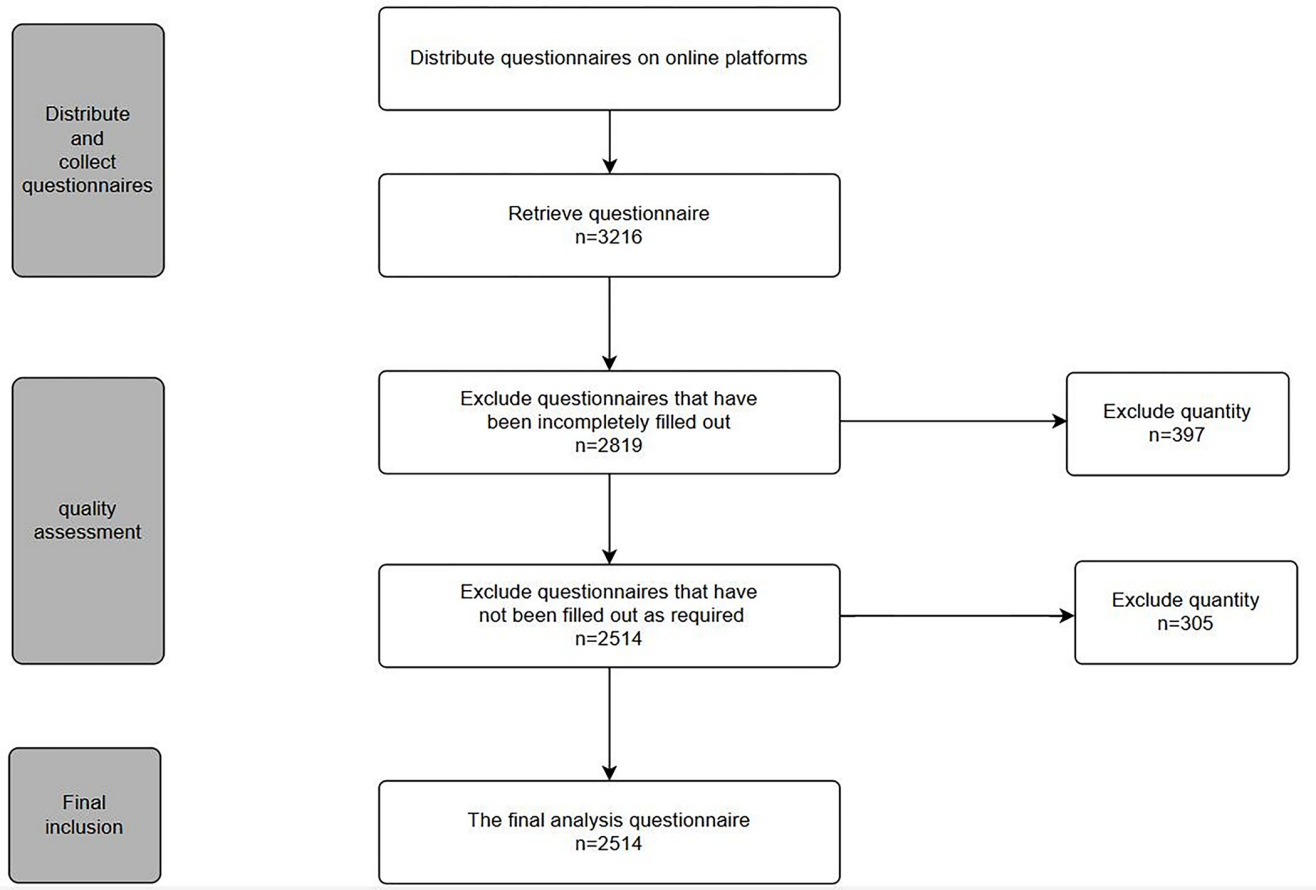
Sample acquisition process.

The sample data for this study were collected online through an online questionnaire platform(https://www.wjx.cn/). A survey questionnaire was distributed to in-service teachers in Chinese TVET school. Through the stages of distributing and collecting questionnaires, quality assessment, and final inclusion, 2514 valid questionnaires were obtained (see Fig 2).

**Fig 3:**
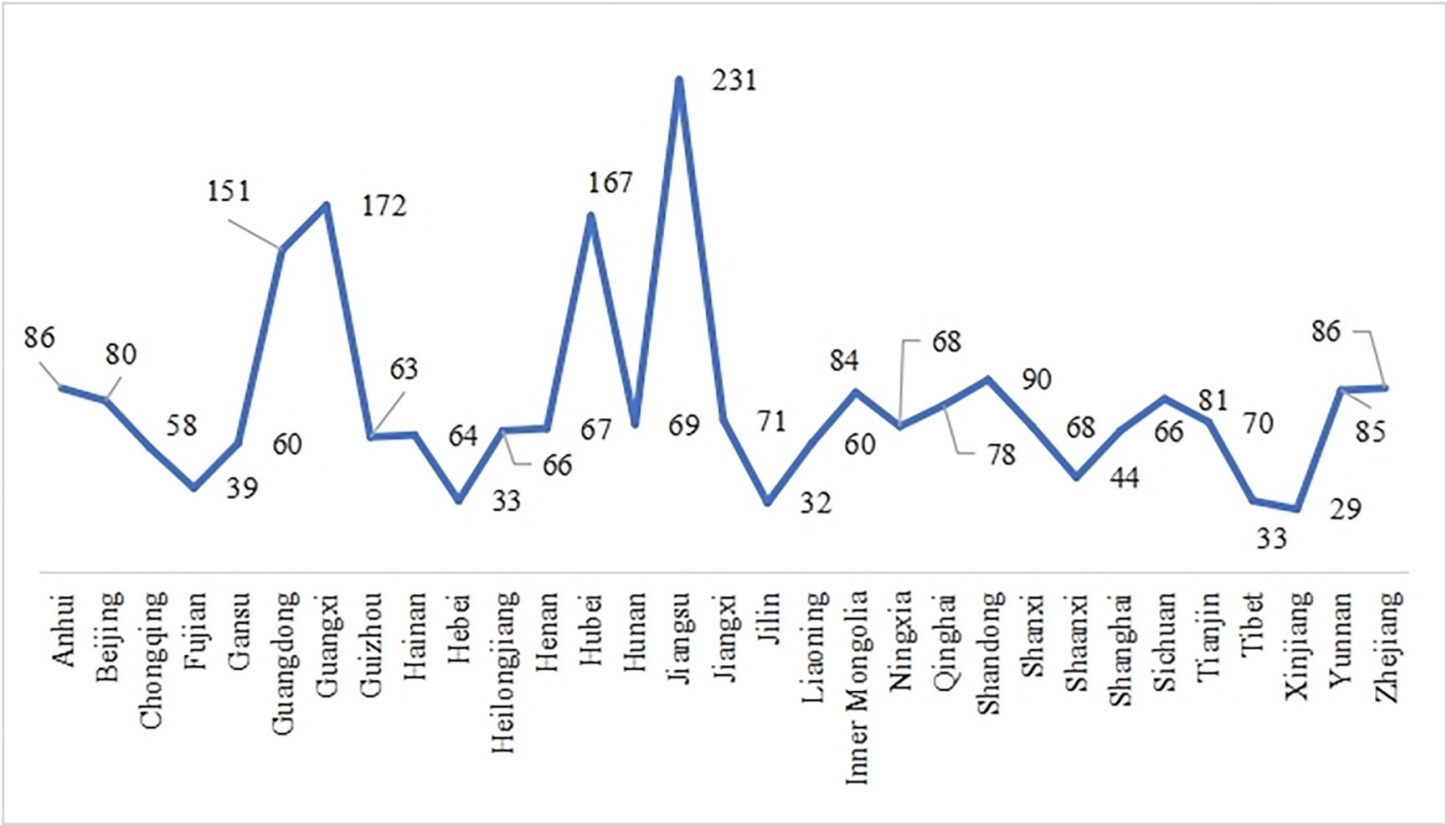
Sample data of provinces.

**Fig 4:**
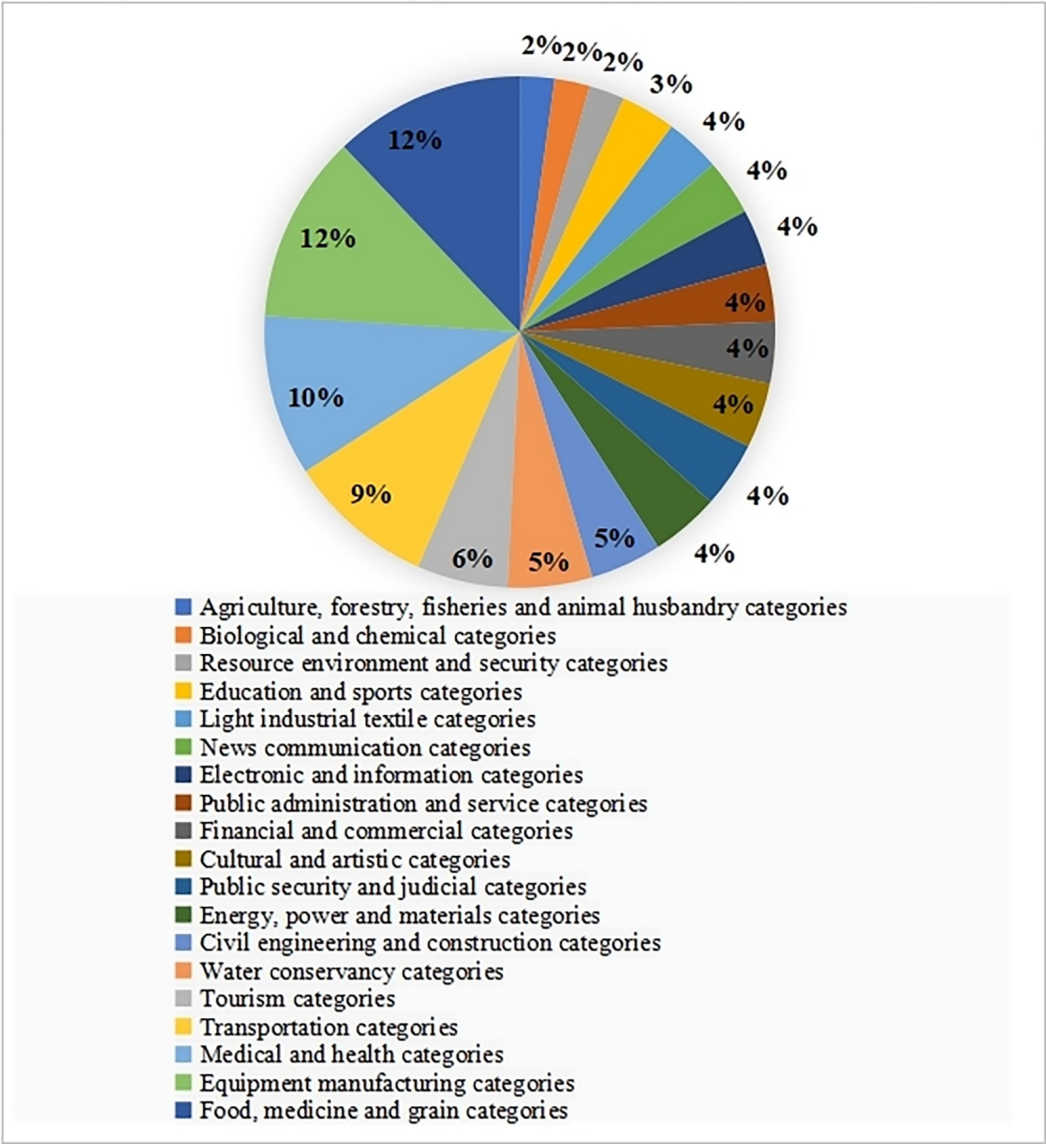
Chinese TVET majors included in the sample.

The participants were distributed across various regions in China, including Jiangsu, Guangdong, Shandong, Zhejiang, Shanghai, Yunnan, Hunan, Xinjiang, Qinghai, Inner Mongolia, Shaanxi, Gansu, Guizhou, Henan, Sichuan, Hebei, Hubei, Anhui, Chongqing, Jiangxi, Ningxia, and more, covering the majority of provinces and regions in China (see Fig 3). The survey respondents represented 19 different TVET majors (Fig 4), ensuring the effectiveness of the questionnaire survey and its ability to provide a reasonably accurate reflection of the current state of digital competence among TVET teachers nationwide.

### 3.2 Assessment tools

The assessment of TVET teachers’ digital competence consists of two main parts. In this section, we will provide an overview of their components.

#### 3.2.1 Personal information

The first part of the online questionnaire collects basic information about the participants. This section is based on factors that influence teacher digital competence, including both internal and external factors. Internal factors include teacher gender, educational level, the type of institution they work for, teacher rank, whether they are "dual-qualified" teachers (commonly recognized as teachers who possess basic educational and vocational qualifications, have expertise in specific vocational principles, and have professional practical abilities, making them competent educators in TVET institutions) [52], years of teaching experience, and more. External factors encompass the level of digitization in the employing institution, whether the teacher has received digital training, and participation in digital competitions, among others. Some factors are represented by multiple questions. Please refer to Table 1 for a detailed breakdown of the basic information collected from the participants.

**Table 1:** The basic information table of the subject teacher.

Category	Option	Number	Percentage
Are ‘dual-qualified’ teachers?	Yes	787	31.3%
	No	1727	68.7%
educational level	Undergraduate level and below	465	18.5%
	Master’s degree graduate	1199	47.69%
	Doctoral degree graduate	850	33.81%
years of work experience	1-4years	207	8.23%
	5-10years	796	31.66%
	11-15years (including 15 years)	1277	50.8%
	More than 15 years	234	9.31%
type of employing institution	Secondary TVET school	386	15.35%
	Higher TVET college (specialized)	1503	59.79%
	Higher TVET university (undergraduate level)	625	24.86%
Teacher’s professional title	Assistant Lecturer (or equivalent title)	356	14.16%
	Lecturer (or equivalent title)	644	25.62%
	Senior Lecturer (or equivalent title)	1269	50.48%
	Principal Senior Lecturer (or equivalent title)	245	9.75%
Whether it is a provincial-level exemplary vocational college	Yes	952	37.87%
	No	1562	62.13%
Whether it is a national-level exemplary vocational college.	Yes	660	26.25%
	No	1854	73.75%
Number of times teachers have received government-organized training on educational information technology capabilities	No	800	31.82%
	1–2 Times	485	19.29%
	3–4 Times	769	30.59%
	5–6 Times	270	10.74%
Winner of the highest-level teaching skills competition	None	606	24.11%
	School-level	159	6.32%
	City-level	1288	51.23%
	Provincial/Regional-level	320	12.73%
	national level	141	5.61%
Number of times teachers have participated in school-based training on educational information technology capabilities	No	987	39.26%
	1–2 Times	536	21.32%
	3–4 Times	817	32.50%
	5–6 Times	143	5.69%
	7 Times and above	31	1.23%

#### 3.2.2 Assessment tool for TVET teachers digital competence

On the basis of the theoretical framework of digital competence for Chinese TVET teachers mentioned earlier, this study follows the empirical approach of "extracting and condensing indicator elements—expert consultation and revision of elements—determining element weights", and ultimately determines that the digital competence model for Chinese technical and vocational education and training teachers includes five major competency domains,: digital mindset and attitude, digital knowledge and skills, digital education and teaching, digital care and support, and digital collaboration and development (Table 2).

**Table 2:** Competency domains and descriptions for vocational college teachers.

Digital Mindset and Attitude	Teachers should embrace fundamental philosophies that adapt to the requirements of the digital era and recognize the importance of digital technology in learning, teaching, and professional practice. They should possess the consciousness and attitude to learn, apply, integrate, and innovate with digital technologies.
Digital Knowledge and Skills:	Teachers need to be proficient digital citizens who can effectively and responsibly acquire, use, develop, and share digital resources. Moreover, they must be skilled in the digital knowledge and technology required for their respective professional domains, as professionals and experts.
Digital Education and Teaching	Teachers should be capable of applying digital technology sensibly in professional teaching, organizing internships and practical training, managing extracurricular activities and classrooms, and using digital formative assessment to conduct dynamic monitoring, continually optimizing the effectiveness of education and teaching.
Digital Care and Support	Teachers should ensure the fairness of acquiring knowledge through digital technology, focus on individualized learning needs, respect students’ individual learning styles, and promote students’ active and effective participation in the digital learning process. They should help students gradually master the digital skills required for their professions and create digital learning products in a standardized manner.
Digital Collaboration and Development	Teachers should use digital technology to communicate and collaborate with communities of interest, proactively update their knowledge structures related to their professions, continuously reflect on and improve their teaching and professional skills, collaborate with businesses to achieve research and innovation, and advance their professional development.

The five major competency domains are further subdivided into 29 specific elements. Within the dimension of "Digital Mindset and Attitude," these elements encompass: Morality, Technology, and Cultivation, Performance Expectations, Effort Expectations, Environmental Influence, Development Attitude, Educational Responsibility. In the dimension of "Digital Knowledge and Skills," the elements include: Digital Knowledge, Digital Devices, software, platforms, Digital Resources, Digital Management. The dimension of "Digital Education and Teaching" is refined into four elements: Digital Teaching Design, Digital Teaching Implementation, Digital Teaching Evaluation, Digital Classroom Management. "Digital Care and Support" comprises four elements: Ensuring Fair Access, Respecting Individual Differences, Promoting Active Participation, Ensuring Effective Use. Within the dimension of "Digital Collaboration and Development," there are four elements: Communication and Collaboration, Keeping Pace with the Forefront, Reflecting and Improving, Research and Innovation.

Based on this framework model, the "Chinese TVET Teacher Digital Competence Self-Assessment Questionnaire" was developed, as presented in Table 3.

**Table 3:** The questionnaire employs a Likert six-point scale, where the average score across all elements represents the digital competence level of the surveyed teachers.

Dimension		Examples of questions	reference
Digital mindset and attitudes	Morality, Technology, and Cultivation	I can guide my own self-cultivation and educate others in the digital environment based on the core values of socialism, emphasizing the comprehensive development of my own professional ethics and technical skills.	[53]
	Performance expectations	I acknowledge the changes that digital technology brings to education and teaching. I am willing to practice to optimize and enhance the quality of teaching, thereby improving work performance.	[54]
	Effort expectations	I can, based on the professional ethics of teaching, utilize digital technology to optimize the effectiveness of education and teaching. I strive to accomplish the dissemination of knowledge, moral education, value guidance, and overall improvement of students’ quality.	[55]
	Environmental influences	I believe in the positive impact of the environment, specifically the ease of accessing digital technology achievements from peers. This positive influence motivates me to actively and consciously enhance my digital skills.	[56]
	Development Attitude	I possess a proactive attitude towards continuously updating my knowledge and enhancing my abilities.	[57]
	Educational responsibility	I understand the value and significance of the digital transformation strategy in reshaping the ecosystem of TVET.	[58]
Digital knowledge and skills	Digital knowledge	I understand the concept of digital teaching and have theoretical knowledge about the equipment, platforms, software, and digital resources required for digital teaching.	[59]
	Digital devices, software, platforms	I am proficient in operating common digital devices related to subject teaching, such as multimedia teaching equipment.	[60]
	Digital resources	I can identify, evaluate, and select digital resources for teaching and learning.	[61]
	Digital management	I am proficient in collecting, managing, processing, and analyzing data, ensuring the privacy of personal information and the security of educational data.	[62]
Digital education and teaching	Digital teaching design	I can use digital technology to analyze students’ learning situations and professional teaching content, and formulate educational objectives.	[63]
	Digital teaching implementation	I can use digital technology to support teaching at different stages and types.	[64]
	Digital teaching evaluation	I understand the evaluation theories related to digital teaching.	[65]
	Digital classroom management	I can use digital technology to conduct combined online and offline educational and moral activities.	[66]
Digital care and support	Ensuring fair access	I can consider and respond to learners’ expectations, levels, and limitations in using digital technology to acquire knowledge.	[67]
	Respect for individuality	I can respect the cognitive characteristics of vocational school students and support different individuals to acquire knowledge and skills asynchronously	[68]
	Promoting active participation	I can use various methods, including digital technology, to activate the connection between learners’ prior knowledge and new knowledge, promoting the establishment of a comprehensive knowledge system.	[69]
	Ensuring effective use	I can ensure that students can search, analyze, and process digital information, enabling them to manage risks and use digital technology responsibly.	[70]
Digital collaboration and development	Communication and collaboration	I can use digital technology to communicate with students, parents, colleagues, and administrators, promoting collaboration in education and teaching.	[71]
	Keeping pace with the forefront	I can use digital technology for self-directed learning, lifelong learning, and enhancing my digital competence.	[72]
	Reflecting and improving	I can use digital technology to document learning and teaching issues, process data, and engage in reflection and improvement.	[73]
	Research and innovation	I can design and develop new teaching resources according to teaching needs, supporting high-quality teaching.	[73]

### 3.3 Data analysis

Upon obtaining all the sample data, this study conducted tests for reliability and validity to determine the effectiveness and practicality of the designed "Chinese TVET teachers Digital Competence Assessment Scale" (RQ1). Subsequently, a test for normal distribution was performed. Given the satisfaction of the normal distribution assumption, IBM SPSS Statistics (27.0) was employed for further data analysis. This analysis encompassed descriptive statistical analysis of the sample data to elucidate the existing state of digital competence among TVET teachers (RQ2). Additionally, it aimed to analyze the factors influencing the digital competence of TVET teachers (RQ3), thereby explaining the disparities in their digital competence.

## 4.Results and analysis

### 4.1 Analysis of the validity of the "Chinese TVET teacher digital competence self-assessment scale"

#### 4.1.1 Reliability test

In this study, Pearson correlation coefficient tests were conducted for each item of the official scale and the total score of teaching competence using SPSS 27.0 software. The results indicated that the Cronbach’s Alpha coefficients for each dimension of digital competence on the scale were greater than 0.7, with an overall reliability of 0.988. This suggests that each item in the questionnaire exhibited a high level of correlation with the overall questionnaire (see Table 4).

**Table 4:** Internal consistency reliability test (N = 2514).

Cronbach’s Alpha	number of items
.988	51

#### 4.1.2 Validation tests

The validation process in this study encompassed both content validity and structural validity.

In terms of content validity, the elements and their descriptions of TVET teachers’ digital competence were established based on the theoretical model and validated through two rounds of verification and revision by 30 experts in the relevant field. Through multiple iterations of analysis, revision, and validation, the descriptions of the model were ensured to be representative. Furthermore, the questionnaire developed based on the digital competence model and its elements received positive feedback during small-scale testing and formal surveys. These results indicate that the survey instrument employed in this study exhibits excellent content validity and aligns with the requirements of this investigation.

Exploratory factor analysis was utilized to examine the structural validity of the assessment scale. To conduct exploratory factor analysis, it is necessary to assess whether the sample data meets the conditions for this type of analysis. The Kaiser-Meyer-Olkin (KMO) measure is a common criterion for this judgment. When the KMO value exceeds 0.6, it indicates that the sample is suitable for exploratory factor analysis [74].

The results revealed a KMO value of 0.964, and Bartlett’s test of sphericity yielded a χ2 value of 12110.107 (with 1128 degrees of freedom), explaining 66.579% of the variance, with a p-value of less than 0.001, indicating significant differences and meeting the conditions for factor analysis. As shown in Table 5, the KMO values for each dimension—digital mindset and attitude, digital knowledge and skills, digital education and teaching, digital care and support, and digital collaboration and development—were all above 0.70. Additionally, all dimensions passed the Bartlett’s test of sphericity (P-value = 0.000<0.05). Therefore, it can be concluded that the variables in this study exhibit high construct validity, indicating that the questionnaire items effectively capture the conceptual information related to the corresponding variables.

**Table 5:** Summary of KMO and bartlett’s sphericity analysis results.

dimension	number of items	KMO value	chi-square value	df	P value
Digital Mindset and Attitudes	7	0.844	767.358	15	0.000**
Digital Knowledge and Skills	9	0.925	1472.667	36	0.000**
Digital Education and Teaching	15	0.945	3258.610	78	0.000**
Digital Care and Support	9	0.922	1687.688	36	0.000**
Digital Collaboration and Development	11	0.947	2586.379	55	0.000**

Note

*p < 0.05

**p < 0.01.

The collected questionnaire data was imported into AMOS 26 software. Upon evaluating the model fit, it was observed that there were no negative error terms, and both standardized coefficients and error terms fell within an acceptable range. This was confirmed through violation estimation checks [75]. The fit indices indicated that the model fit the data well. The chi-square to degrees of freedom ratio was 3.330, which is less than 5, indicating a good fit. The Goodness of Fit Index (GFI) was 0.818, close to 0.9. In the context of the complexity of social and psychological issues, a value slightly below 0.9 (but above 0.8) is generally considered acceptable [76].

The Root Mean Square Error of Approximation (RMSEA) was 0.091, which is less than 0.10, signifying a reasonable fit. Additionally, the Comparative Fit Index (CFI) was 0.915, the Incremental Fit Index (IFI) was 0.916, and the Tucker-Lewis Index (TLI) was 0.902, all exceeding 0.9 (as shown in Table 6). These values indicate excellent fit indices, suggesting a well-fitted model. The high correlations among dimensions imply a comprehensive representation of the competencies that teachers should possess in the digital age.

**Table 6:** Confirmatory factor analysis valuescommon indicators.

	χ^2^	df	chi-square degrees of freedom ratioχ^2^/df	GFI	RMSEA	CFI	IFI	TLI
criteria for judgment	-	-	<5	>0.9	<0.10	>0.9	>0.9	>0.9
overall dimension values	662.683	199	3.330	0.818 (approaching0.9)	0.091	0.915	0.916	0.902
model fit assessment	passed	passed	passed	passed	passed	passed	passed	passed

### 4.2 Overall digital competence level of TVET teachers Nationwide

Examining the overall data from 2514 samples nationwide, the average score for TVET teachers’ digital competence was M = 4.19with a standard deviation of SD = 0.756, falling between "somewhat fitting" and "fairly fitting." Among these, 836 TVET teachers scored above the average, constituting 33.25% of the sample. Conversely, 1677 teachers scored below the average, making up 66.70% of the sample. Notably, 1538 individuals fell into the "somewhat fitting" or below category, accounting for 61.17%.

Examining each dimension, the mean scores ranked as follows: digital collaboration and development > digital education and teaching > digital care and support > digital mindset and attitude > digital knowledge and skills. In specific terms, 942 individuals (37.42%) scored above the average in the dimension of digital collaboration and development, while 1572 individuals (62.52%) scored below the average. Additionally, 884 individuals (35.16%) scored above the average in digital education and teaching, with 1630 individuals (64.83%) scoring below. In the dimension of digital care and support, 887 individuals (35.28%) scored above the average, and 1627 individuals (64.71%) scored below. Notably, in the dimensions of digital education and teaching, digital collaboration and development, and digital care and support, the teachers exhibited optimistic tendencies. However, in the dimensions of digital mindset and attitude and digital knowledge and skills, a significant portion fell into the "somewhat fitting" or below category, constituting 59.94% and 61.05%, respectively (see Table 7).

**Table 7:** Basic overview of TVET teachers’ digital competence.

aspect	Mean	Standard deviation	N1 (Above the average score.)	N2 (Below the average score.)	N3 (Below 4 points.)
total score of digital competence	4.19	0.756	836 (33.25%)	1677 (66.70%)	1538 (61.17%)
Digital Mindset and Attitude	4.14	0.96	871 (34.64%)	1642 (65.31%)	1507 (59.94%)
digital knowledge and skills	4.12	0.79	839 (33.37%)	1674 (66.58%)	1535 (61.05%)
digital education and teaching	4.23	0.73	884 (35.16%)	1630 (64.83%)	1027 (40.85%)
digital care and support	4.21	0.72	887 (35.28%)	1627 (64.71%)	1097 (43.63%)
digital collaboration and development	4.25	0.73	942 (37.47%)	1572 (62.52%)	1008 (40.09%)

**Fig 5:**
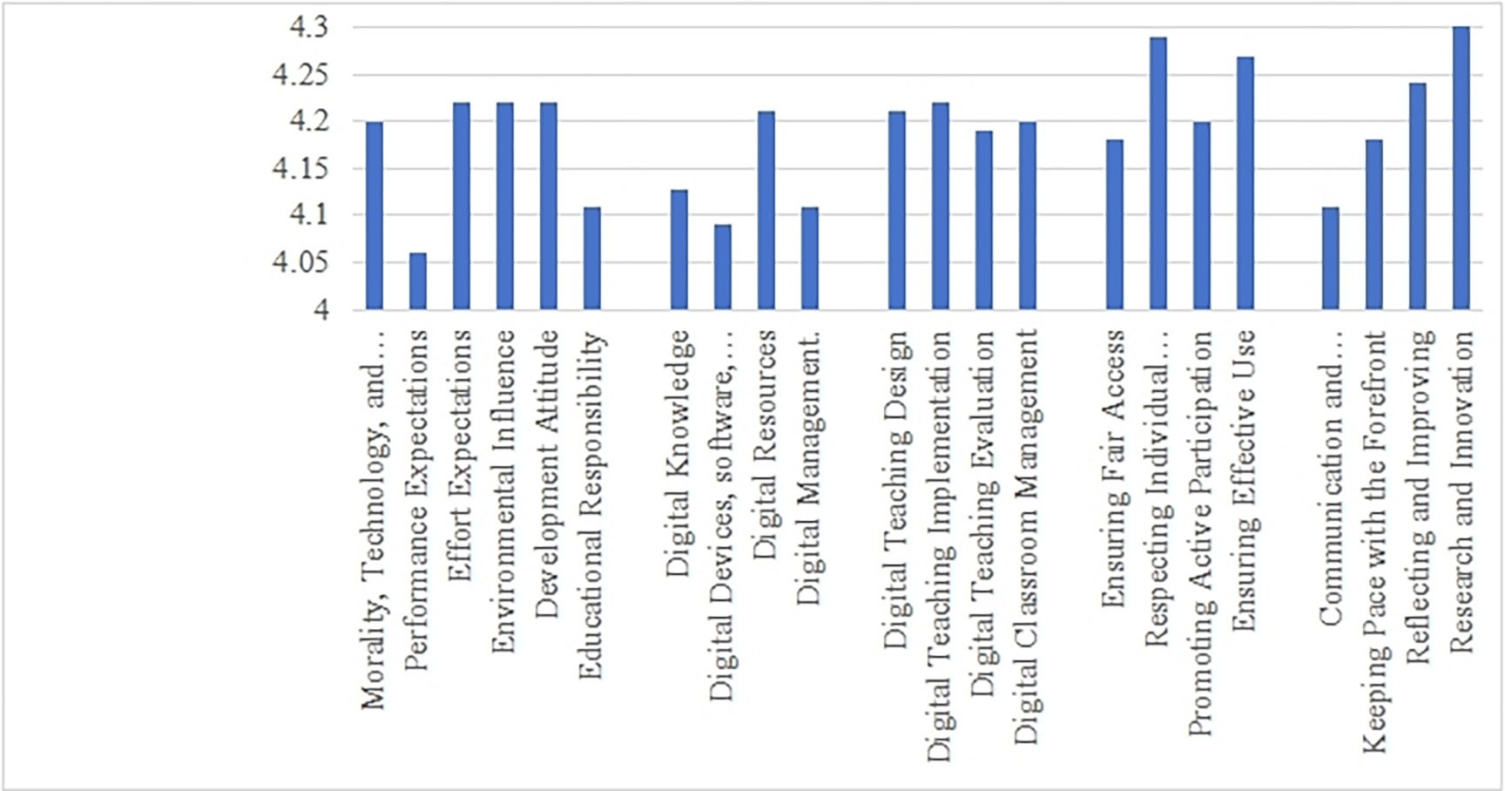
Scores of various indicators of digital competence for TVET teachers.

Looking at specific indicators, in the dimension of digital mindset and attitude, nationally, environmental influence and effort expectations scored the highest, followed by moral and technical cultivation, development attitude, and educational responsibility. Performance expectations scored the lowest, making it the lowest-scoring indicator among all dimensions. In the dimension of digital knowledge and skills, the highest-scoring indicator was digital resources, followed by digital management and knowledge, while digital devices, software, and platforms scored the lowest. In digital education and teaching, the scores ranked as digital teaching implementation > digital teaching design > digital classroom management > digital teaching evaluation. In the dimension of digital care and support, the highest-scoring indicator was respecting individual differences, making it the highest-scoring indicator overall, while ensuring equal access scored the lowest. Finally, in the dimension of digital collaboration and development, the indicator with the lowest score was collaborative communication, while research and innovation scored the highest, as shown in Fig 5.

Following the categorization standards from the official website of the National Bureau of Statistics of China, the provinces involved in this study were divided into three major economic regions: Eastern, Central, and Western. The Eastern region includes 11 provincial-level administrative units: Beijing, Tianjin, Hebei, Shanghai, Liaoning, Jiangsu, Zhejiang, Shandong, Fujian, Guangdong, and Hainan. The Central region comprises 8 provincial-level administrative units: Shanxi, Anhui, Jiangxi, Henan, Hubei, Hunan, Heilongjiang, and Jilin. The Western region includes 12 provincial-level administrative units: Sichuan, Guizhou, Chongqing, Yunnan, Gansu, Shaanxi, Ningxia, Qinghai, Xinjiang, Inner Mongolia, Tibet, and Guangxi.

**Fig 6:**
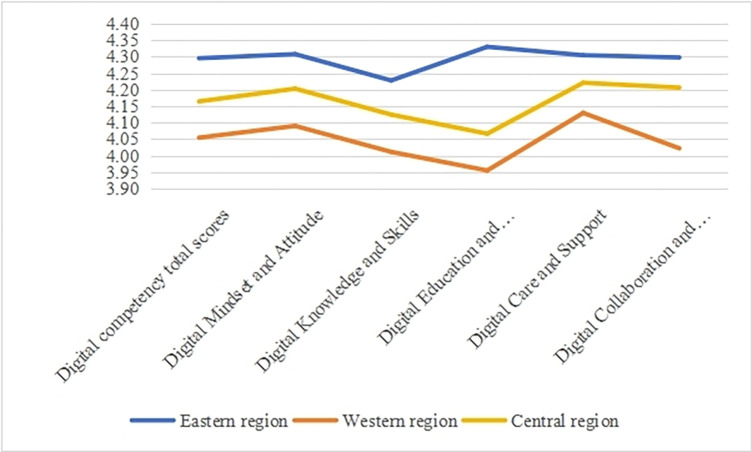
Scores of digital competence for TVET teachers in the Eastern and Western regions.

By analyzing the total scores and dimensional scores of digital competence among TVET teachers in Eastern, Central, and Western regions, a graph (as shown in Fig 6) was generated. The graph illustrates that the Digital Competence Total Scores of teachers in the Eastern region were significantly higher than those in the Central and Western regions. Additionally, examining the scores by individual dimensions, a consistent pattern emerged, revealing the characteristic of "higher in the East, intermediate in the Central, and lower in the West" concerning TVET teachers’ digital competence.

TVET’s digital development is closely related to regional economic development. The digitization of TVET is influenced by regional market economy adjustments to the layout and resource allocation of TVET industries. High-quality digital educational resources, the creation of a digital infrastructure, and the construction of digital educational hardware facilities tend to converge in economically developed areas. Currently, China’s economic development exhibits regional imbalances, with a trend of Eastern region > Central region > Western region [77]. This trend aligns with the developmental characteristics observed in this study: higher digital competence levels in the Eastern region compared to the Central and Western regions.

### 4.3 The differences in the impact of internal and external factors on TVET teachers’ digital competence

The factors designed in this study to influence the digital competence level of TVET teachers are categorized into internal and external factors. The correlation results indicate that all factors, except gender, show varying degrees of significance in relation to the Digital Competence Total Score. Significant relationships also exist with certain dimensions, as shown in Table 8.

**Table 8:** Results of correlation analysis between internal and external factors and TVET teachers’ digital competence.

	Digital Competence Total Score	Digital Mindset and Attitude	digital knowledge and skills	digital education and teaching	digital care and support	digital collaboration and development
academic qualification	-0.496**	-0.517**	-0.509**	-0.478**	-0.445**	-0.417**
type of employing institution	-0.168**	-0.304**	-0.238**	-0.099**	-0.109**	-0.094**
professional title	-0.144**	-0.198**	-0.175**	-0.087**	-0.137**	-0.121**
dual-qualified teachers	-0.573**	-0.614**	-0.542**	-0.554**	-0.514**	-0.504**
years of work experience	-0.119**	-0.103**	-0.153**	-0.106**	-0.119**	-0.094**
digitalization level of the employing institution	-0.115**	-0.007	-0.066**	-0.157**	-0.146**	-0.141**
participation in digital competitions	0.050*	0.051*	0.070**	0.031	0.049*	0.046*
received digital training	-0.489**	-0.582**	-0.507**	-0.410**	-0.462**	-0.411**

Note

*p < 0.05

**p < 0.01.

#### 4.3.1. Internal factors, excluding gender, have significant differences in impact on the digital competence level of TVET teachers

According to the analysis results, teachers with undergraduate degrees and below exhibit the highest overall digital competence, followed by those with master’s and doctoral degrees. However, when looking at individual dimensions, teachers with master’s and doctoral degrees score significantly higher than those with undergraduate degrees in dimensions such as digital mindset and attitudes, and digital knowledge and skills (see Table 9). This suggests that TVET teachers who have received higher levels of education generally exhibit improved digital mindsets, attitudes, knowledge, and skills. This improvement is largely attributed to a series of measures outlined in China’s Education Informatization 2.0 Action Plan aimed at enhancing the level of educational informatization application and information literacy of both teachers and students.

**Table 9:** Differential analysis of teachers’ educational background and digital competence levels.

	Educational Background Types (Mean ± Standard Deviation)		
	Bachelor’s Degree and Below	Master’s Graduate	Doctoral Graduate
Digital Mindset and Attitude	4.17±0.79	4.74±0.98	5.16±0.56
Digital Knowledge and Skills	4.15±0.80	4.77±0.73	4.53±0.51
Digital Education and Teaching	4.64±0.83	3.98±0.66	3.71±0.46
Digital Care and Support	3.78±0.88	4.07±0.64	4.20±0.49
Digital Collaboration and Development	3.60±0.93	4.29±0.64	4.39±0.49
Digital Competence Total Score	4.70±0.80	4.72±0.67	4.39±0.50

However, it is observed that teachers with higher academic qualifications perform significantly worse in dimensions such as digital education and teaching, indicating that specific training and support related to the practical transformation of digital education theories into classroom practices are lacking for teachers with master’s and doctoral degrees. This gap results in a theoretical understanding without practical implementation, making it difficult for teachers with higher academic qualifications to establish a beneficial interactive relationship between digital teaching theories and the practical aspects of digital education and teaching.

Vocational colleges are categorized into three types: secondary TVET schools, higher vocational colleges (specialized), and higher vocational colleges (undergraduate). Secondary TVET schools scored above 4 points in both the Digital Competence Total Score and individual dimensions, making them the best-performing among the three types of vocational colleges (see Table 10). Following are the higher vocational colleges (specialized), while higher vocational colleges (undergraduate) scored relatively lower. Particularly, in the dimensions of digital mindset and attitude and digital knowledge and skills, the scores of higher vocational colleges (undergraduate) were significantly lower than the other two types of colleges. This indicates that secondary TVET schools and higher vocational colleges (specialized) are currently in a stable development stage regarding the digital competence of their teachers. In contrast, higher vocational colleges (undergraduate) are still in the experimental phase, representing a nascent aspect of TVET reform. The development of digital competence among teachers in higher vocational colleges (undergraduate) needs to progress steadily based on the foundation of their stable development to ensure successful implementation.

**Table 10:** Differential analysis of digital competence total scores based on the type of employing institution for teachers.

	Type of Employing Institution (Mean ± Standard Deviation)		
	Secondary TVET School	Higher TVET College (Specialized)	Higher TVET University (Undergraduate)
Digital Mindset and Attitude	4.81±1.08	4.12±0.93	3.82±0.73
Digital Knowledge and Skills	4.55±1.03	4.12±0.74	3.92±0.63
Digital Education and Teaching	4.38±1.07	4.20±0.70	4.13±0.54
Digital Care and Support	4.44±1.05	4.21±0.68	4.16±0.53
Digital Collaboration and Development	4.40±1.07	4.21±0.68	4.16±0.56
Digital Competence Total Score	4.48±1.01	4.18±0.70	4.06±0.55

In the dimension of digital mindset and attitude, teachers with the "Dual-Expertise" title scored significantly higher than their non-"Dual-Expertise" counterparts. Moreover, in other dimensions as well as the total score, "Dual-Expertise" teachers outperformed their non-"Dual-Expertise" peers (see Table 11). This indicates that TVET teachers with the "Dual-Expertise" title exhibit stronger digital survival and adaptation capabilities, educational research skills, continuous professional development, and innovation in educational practices. The continuous promotion of the certification of "Dual-Expertise Teachers" holds significant importance in enhancing the digital competence of TVET teachers.

**Table 11:** Differential analysis of dual-qualified teachers and digital competence total scores.

	Whether Belongs to ’Dual-Qualified’ Teachers (Mean ± Standard Deviation)	
	Yes	No
Digital Mindset and Attitude	5.02±0.91	3.75±0.68
Digital Knowledge and Skills	4.78±0.83	3.85±0.58
Digital Education and Teaching	4.82±0.76	3.93±0.54
Digital Care and Support	4.79±0.74	3.98±0.56
Digital Collaboration and Development	4.77±0.77	3.98±0.56
Digital Competence Total Score	4.82±0.73	3.91±0.54

In this study, teaching experience was categorized into four groups: 1–4 years, 5–10 years, 11–15 years, and over 15 years (see Table 12). The results indicated a U-shaped developmental trend between teaching experience and digital competence level. As teaching experience increases, teachers’ expertise gradually becomes richer, evolving from reliance on experience to the formation of practical knowledge and the integration of theory and practice. This progression is essential for becoming a proficient teacher and represents the significance and value that the accumulation of work experience brings to teachers at different stages of their professional development.

**Table 12:** Differential analysis of teacher’s years of work experience and digital competence total scores.

	Years of Work Experience (Mean ± Standard Deviation)			
	1–4 years	5–10 years	11–15 years (including 15 years)	More than 15 years
Digital Mindset and Attitude	5.03±0.68	4.25±1.01	3.74±0.69	5.26±0.78
Digital Knowledge and Skills	4.87±0.65	4.27±0.77	3.80±0.59	4.89±0.84
Digital Education and Teaching	4.74±0.68	4.28±0.82	3.98±0.52	4.75±0.91
Digital Care and Support	4.81±0.77	4.32±0.76	3.98±0.51	4.83±0.86
Digital Collaboration and Development	4.80±0.72	4.24±0.80	4.04±0.53	4.71±0.95
Digital Competence Total Score	4.83±0.65	4.27±0.80	3.93±0.52	4.85±0.81

However, the study also found that the performance of teachers with longer teaching experience is not necessarily superior to that of their less experienced counterparts, especially in dimensions such as digital mindset and attitude, and digital knowledge and skills. This discrepancy might arise from experienced teachers overly relying on teaching experience and neglecting the theoretical aspects of education, leading to a conflict between practical knowledge and theoretical understanding, which could result in professional burnout [78]. Particularly, for teachers with over 15 years of experience or those nearing retirement, they might find it challenging to keep up with and master the emerging digital competencies demanded by digital education [79]. They might inadvertently overlook the centrality of students, reducing their effectiveness in providing adequate digital care and guidance for students’ career development.

On the contrary, less experienced teachers, especially in their early careers, tend to invest high levels of effort, are enthusiastic about their work, and possess high psychological capital. They are less prone to experiencing professional burnout. Even if they face challenges or lower effectiveness in the short term, they can attribute these issues to specific causes, remain closely engaged with the dynamic development of students’ digital capabilities, and proactively address problems arising from digital teaching practices.

The results of the analysis of variance revealed that Lecturers (or those with equivalent titles) had the highest overall digital competence scores and scores in individual dimensions. They were followed by Assistant Lecturers (or those with equivalent titles). In contrast, Senior Lecturers (or those with equivalent titles) and Professors (or those with equivalent titles) scored lower, with their competence levels decreasing as their academic titles advanced, presenting an inverted U-shaped pattern (see Table 13). This suggests that the digital competence of TVET teachers at the senior and higher levels of academic titles is not optimal. Specifically, in the dimensions of digital mindset and attitude, and digital knowledge and skills, Senior Lecturers (or those with equivalent titles) and Professors (or those with equivalent titles) scored much lower than Lecturers (or those with equivalent titles) and Assistant Lecturers (or those with equivalent titles).

**Table 13:** Differential analysis of teacher’s professional title type and digital competence total scores.

	Teacher’s Professional Title Type (Mean ± Standard Deviation)			
	Assistant Lecturer (or equivalent title)	Lecturer (or equivalent title)	Senior Lecturer (or equivalent title)	Principal Lecturer (or equivalent title)
Digital Mindset and Attitude	4.30±1.17	4.53±0.99	3.95±0.81	3.94±0.94
Digital Knowledge and Skills	4.23±1.04	4.43±0.84	4.01±0.63	3.95±0.78
Digital Education and Teaching	4.29±0.91	4.38±0.89	4.07±0.60	4.34±0.51
Digital Care and Support	4.37±0.97	4.40±0.83	4.12±0.57	4.19±0.63
Digital Collaboration and Development	4.33±0.92	4.43±0.86	4.09±0.59	4.28±0.58
Digital Competence Total Score	4.30±0.94	4.42±0.84	4.06±0.59	4.18±0.62

However, teachers with higher academic titles performed relatively better in digital education and teaching, as well as digital collaboration and development, indicating the promoting role of educational experience in teachers’ competence development. The decline in comprehensive competence among teachers at higher academic levels might be attributed to the increasing responsibilities that come with higher academic titles. As teachers advance in their careers, they often take on additional administrative roles alongside their teaching duties. This dispersion of focus may lead to neglect of the actual teaching process and disregard for the central role of students. The declining ability of teachers to provide digital care and support as their academic titles rise, as observed in this study, precisely reflects this phenomenon.

#### 4.3.2 Impact of external factors on the digital competence of TVET teachers

External factors in this study were measured by the level of digitalization in teachers’ employing institutions, evaluated through two criteria: "Is the institution a provincial-level exemplary vocational college?" and "Is the institution a national-level exemplary vocational college?" The results indicated that the higher the level of digitalization in the employing institution (being either a national or provincial exemplary vocational college), the higher the digital competence scores of teachers in both total and individual dimensions. On average, teachers working in national-level exemplary vocational colleges exhibited higher digital competence levels in all dimensions compared to those in provincial-level exemplary vocational colleges. This suggests that institutions recognized as provincial or national exemplary vocational colleges maintain superior overall educational standards, faculty quality, educational resources, and teaching levels. For teachers in such institutions, the external professional system they are part of is more stable and comprehensive, fostering a "chain reaction" and "radiation effect" that indirectly promotes the development of their digital competence.

**Fig 7:**
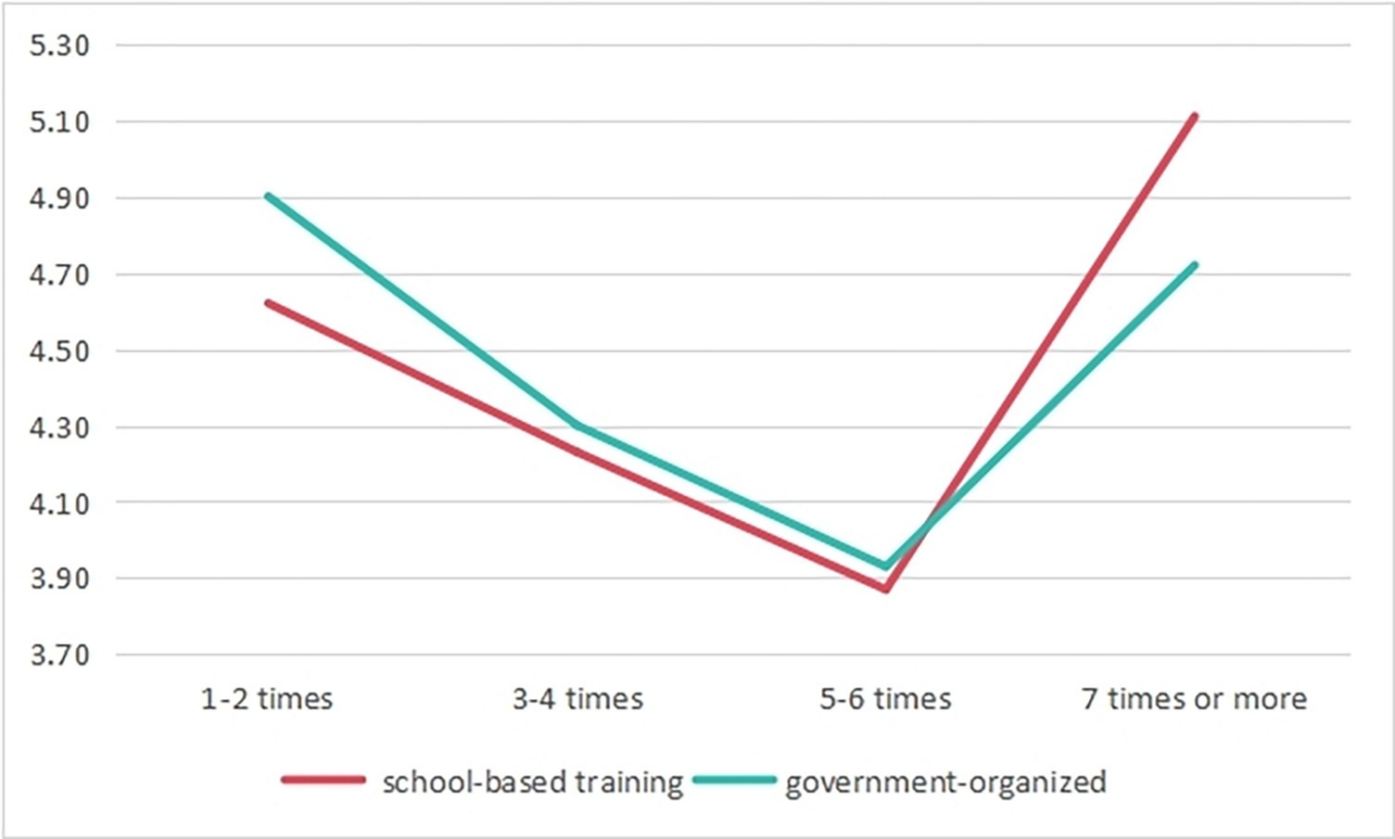
Comparative chart of digital competence improvement among TVET teachers participating in different types of digital training.

Regarding digital training, teachers’ participation was categorized into government-organized and school-based training. The analysis demonstrated that teachers who had undergone digital training displayed significantly higher digital competence compared to those who hadn’t. Furthermore, the study segmented teachers’ training participation into categories: 1–2 times, 3–4 times, 5–6 times, and 7 times or more. The results indicated a significant main effect of training frequency on teachers’ overall competence and various dimensions. As the training frequency increased, teachers’ digital competence showed a declining trend, particularly among those participating in government-organized training. However, when the training frequency reached 7 times or more, there was a significant improvement in the mean digital competence scores. Interestingly, teachers who participated in school-based training exhibited a more pronounced increase compared to those involved in government-organized training (see Fig 7). This indicates that short-term participation in digital training might not immediately enhance teachers’ digital competence; however, it has a significant positive long-term impact, especially in the case of school-based training, which proves to be more effective than government-organized training.

Additionally, teachers who participated in teaching (skills) competitions demonstrated higher digital competence scores than those who did not. Examining the types of competitions, the scores followed the pattern of provincial (regional) > school-level > national > municipal level, indicating that provincial (regional) level competitions had the most significant positive impact on teachers’ digital competence. Refer to Table 14 for detailed information.

**Table 14:** Differential analysis of external factors and digital competence total scores of teachers.

Is it a provincial demonstration vocational school?	Digital Competence Total Score	
	M.	S.D.
Yes	4.60	1.05
No	4.05	0.75
Unclear	4.06	0.55
Is it a national demonstration vocational school?	Digital Competence Total Score	
	M.	S.D.
Yes	4.62	0.69
No	3.88	0.68
Unclear	4.34	0.70
Highest Award in Teaching Skills Competition	Digital Competence Total Score	
	M.	S.D.
None	4.18	0.83
School Level	4.93	0.65
City/District Level	3.93	0.38
Provincial/Regional Level	5.00	0.66
National Level	4.01	1.15
Participation in Government-Organized Training on Educational Informatization	Digital Competence Total Score	
	M.	S.D.
Yes	4.38	0.84
No	4.03	0.58
Has the individual attended the educational informatization training organized by the school?	Digital Competence Total Score	
	M.	S.D.
Yes	4.39	0.87
No	4.01	0.53

## 5.Discussion

This study aimed to develop a framework tool tailored to measure the digital competence of TVET teachers in China. The results indicated that the current digital competence level of TVET teachers in China is moderate to low.

Among the various dimensions of digital competence, the ability to provide digital care and support received the highest scores. Within this ability, the indicator "respecting individual differences" scored particularly high. This aligns with the core role of digital technology in education advocated by the Chinese government—to promote the concept of educational equity. In addition, among the single dimensional impact indicators of digital competence, the scores of digital mindset and attitudes, digital knowledge and skills dimensions are relatively low, which is consistent with the study [39]. In the theoretical model of digital competence for vocational education teachers, digital mindset and attitudes represent the "attitude" axis, which is the mapping of implicit competence in the digital competence of vocational education teachers. It plays a long-term and continuous optimization and adjustment role in the development of explicit competence. From the perspective of professional development of vocational education teachers, the cultivation of digital concepts and attitudes should be leading and continuously developing. Therefore, leading teachers to form intelligent teaching personalities, beliefs, and consciousness, strengthening the perception of digital competence value, and teachers identifying with the trend of educational digitalization development and its value to students, their digital competence improvement is more likely to be influenced by autonomous growth motivation and external regulatory motivation [39].

In the dimension of digital education and teaching, the indicator "implementation of digital teaching" received the highest score, while "digital teaching evaluation" received the lowest. This could be attributed to teachers commonly engaging in selecting and organizing digital resources to assist teaching, which is a common activity. On the other hand, digital teaching evaluation, often conducted through methods like exam rankings, rarely incorporates digital technology-driven assessment [80].This indicates that the cultivation of abilities should further transform into practical fields, building digital education practice venues for teachers, improving digital education teaching equipment, establishing digital education data platforms, training teachers in digital teaching application abilities, and bridging the gap between digital education theory and practical abilities.

Furthermore, the regional analysis showed that TVET teachers in the eastern region of China demonstrated significantly higher digital competence compared to their counterparts in the central and western regions. This discrepancy can be directly related to the role of economic development levels in the eastern, central, and western regions in regulating the layout and resource allocation of TVET industries. This finding is consistent with the results of studies by some scholars [81].

The study revealed significant differences in teachers’ digital competence related to factors other than gender. Concerning educational qualifications, the study confirmed that teachers with higher educational qualifications had lower digital competence. This finding seems contradictory to common beliefs. A possible explanation could be that individuals with higher educational qualifications tend to be more self-restrained. They possess higher levels of digital technology awareness, leading them to be humbler about their abilities. The study found a U-shaped relationship between work experience and teachers’ digital competence. In other words, teachers who are newly employed and those with extensive work experience show better development in their abilities, while those with 5–10 years of work experience exhibit poorer development. This finding is similar to research conducted by others [82]. Comprehensive ability development has become the core of pre-service teacher education. Teachers with extensive work experience can identify effective digital resources to assist in teaching, enhancing their digital competence. Regarding professional titles, teachers’ digital competence demonstrated an inverted U-shaped relationship. A possible explanation is that increasing job responsibilities make it challenging for teachers to focus entirely on student development. Therefore, in the process of cultivating teachers’ digital competence, we should improve the digital teaching and research support system, achieve organizational support for teacher groups, and build a teacher team consisting of novice teachers, research-oriented teachers, expert teachers, TVET experts based on the teaching group. We should carry out continuous training and design targeted training programs for different teachers’ digital competence attributes. For example, the cultivation of teachers’ digital knowledge can be achieved through providing personalized learning resources to achieve self-directed learning, and the cultivation of teachers’ skills can be achieved through cooperative teacher practice projects and training projects. The personality, consciousness, and beliefs of teachers need to be developed through long-term and continuous teacher training plans, rather than short-term teacher exchanges, Provide targeted training to address the differences in digital competency development among teachers with different educational levels, ages, and professional titles. In addition, a dynamic evaluation mechanism for teachers’ digital competence will be designed, and statistical analysis methods will be used to classify teachers’ digital competence into different levels. Training interventions will be provided for low-level teachers, and advanced reinforcement will be implemented for middle-level teachers. High level teachers will be ensured to have sustainable development and promote collaborative and mutual assistance among low-level and middle-level teachers.

Teachers who have undergone digital training exhibited higher digital competence than those who haven’t. However, rapid improvements in efficiency are challenging in the short term, a finding consistent with the results of research by other scholars [83]. It should be emphasized that increasing the quantity of digital competency training and improving the quality of training should be equally important. Starting from the early, middle, and later stages of training, we should strengthen the needs analysis before training, pay attention to the diversity and periodicity of content and form in the middle stage of training, and finally strengthen the evaluation feedback in the later stage of training to achieve visualization of training effects and maximize training effectiveness. Teachers who participated in digital (skills) competitions showed significant differences in digital competence. The more competitions they participated in, the higher their digital competence. However, different types of competition led to different improvement effects. Provincial (regional) level competitions had the best effect, possibly because teachers in the same region have consistent or similar educational development foundations. In this context, teachers can better leverage their strengths. Continuous participation in formal or informal activities that promote teacher development is crucial for enhancing digital competence [84,85]

This study confirms that teacher digital competence is the result of multiple integrated factors, which work together and influence each other internally. Therefore, in the future, it is necessary to promote the development of digital competence of vocational education teachers through the coordinated development of the system as a whole. Based on the ecological changes in the digital transformation of TVET, a new overall layout plan and practical action plan for the development of digital competence of vocational education teachers should be formulated, and the supply and services of the digital transformation system for TVET should be optimized, so that there are feasible rules and regulations for the digital transformation of TVET and the development of digital competence of the teacher group. In addition, an incentive and guarantee system for enhancing the digital competence of vocational education teachers should be established. The enhancement of teachers’ digital competence requires a significant investment of manpower, material resources, and financial resources. A special funding mechanism for digital competence enhancement should be jointly funded by the government, industry enterprises, universities, and social organizations to ensure the economic support for teachers’ ability enhancement.

## 6.Conclusions

This study aimed to develop a measurement tool for assessing the digital competence of TVET teachers in China and to explore the factors influencing their digital competence. To achieve this goal, the study utilized industry standards on "Teacher Digital Literacy" released by the Chinese Ministry of Education and "Digital Campus Norms for Vocational Colleges" as the basis for developing a self-assessment tool. This tool was then applied to 2514 in-service TVET teachers in China. The results showed that the tool is an effective and reliable instrument for measuring the digital competence of TVET teachers. Subsequently, the study applied one-way (variance) analysis and multiple linear regression. The results emphasized that, except for gender, all other internal and external factors were significantly related to teachers’ digital competence. Therefore, to enable teachers to become experts in using digital technology to enhance teaching and improve students’ digital competence, it is necessary to carefully plan teacher development based on different aspects of digital competence (i.e., competence areas). Additionally, when developing plans aimed at increasing teachers’ digital competence, it is essential to consider setting different training paths based on different conditions, such as educational qualifications, professional titles, and work experience, to meet the diverse development needs of teachers.

However, this study has its limitations. First, the sample size of the current situation survey needs to be increased. The study distributed questionnaires online to vocational college teachers from 31 provinces (excluding Hong Kong, Macao, and Taiwan), with a total of 2514 valid responses. While this sample broadly reflects the ability levels of teachers at different levels, compared to the total of 1.29 million full-time teachers in vocational schools nationwide, the sample size is relatively small. Future research should expand the sample size to obtain a more comprehensive understanding of the digital competence levels of TVET teachers in China. Additionally, this study relied on teachers’ self-perception questionnaires rather than objective evaluations of digital competence. Subjectivity in evaluation is inevitable, and it may not accurately reflect the true level of teachers’ current digital competence. Future research could combine teacher self-assessment with third-party evaluations to provide a more objective reflection of the digital competence levels of TVET teachers in China.

## References

[1] Office of Educaitonal Technology. Teacher Digital Learning Guide[R]. U.S.: Department of Education, 2021.

[2] Office of Educaitonal Technology. School Leader Digital Learning Guide [EB/OL]. U.S.: Department of Education,2021.

[3] Ministère de l’Education Nationale et de la Jeunesse.12 mesures pour faire du lycée professionnel un choix d’avenir pour les jeunes et les entreprises[EB/OL]. (2023-05-04) [2023-08-03]. https://www.education.gouv.fr/12-mesures-pour-faire-du-lycee-professionnel-un-choix-davenir-pour-les-jeunes-et-les-entreprises-378032.

[4] Department for Digital, Culture, Media & Sport. National Data Strategy [EB/OL].(2020-12-09)[2022-03-28].https://www.gov.uk/government/publications/uk-national-data-strategy/national-data-strategy.

[5] UNESCO. (2018). UNESCO ICT competency framework for teachers (Vol. 2018, p. 60). París: UNESCO. Retrieved from https://unesdoc.unesco.org/ark:/48223/ pf0000265721.

[6] Redecker C. European Framework for the Digital Competence of Educators: DigCompEdu. 2017. https://joint-research-centre.ec.europa.eu/digcompedu_en#:~:text=The%20European%20Framework%20for%20the%20Digital%20Competence%20of,the%20development%20of%20educator-specific%20digital%20competences%20in%20Europe

[7] Koehler M. J., Mishra P., & Cain W. (2013). What is Technological Pedagogical Content Knowledge (TPACK)? Journal of Education, 193(3), 13–19. 10.1177/002205741319300303

[8] Janssen J., Stoyanov S., Ferrari A., Punie Y., Pannekeet K. & Sloep P. (2013). Experts’ views on digital competence: commonalities and differences. Computers & Education, 68 (oct.), 473–481.

[9] UNESCO. Digital skills development in TVET teacher training. 2020. https://unevoc.unesco.org/pub/trends_mapping_study_digital_skills_development_in_tvet_teacher_training.pdf.

[10] Youmei Wang, Dan Wang, Weiyi Liang, et al. From International Experience to Local Practice: A Breakthrough Method for the Digital Transformation of Technical and Vocational Education and Training in China. China Vocational and Technical Education, 2024, (06): 49–58+65.

[11] Ministry of Education of the People’s Republic of China. Notice from the Ministry of Education on Issuing the Education Industry Standard for Teacher Digital Literacy [EB/OL] (2022-11-30) [2023-06-20]. http://www.moe.gov.cn/srcsite/A16/s3342/202302/t20230214_1044634.html.

[12] Institute of Education, Tsinghua University et al. Vocational Education Informatization Development Report (2021 edition). 2022.07. https://xyzyzdw.stiei.edu.cn/_upload/article/files/1a/2c/d14242664794bccc0dce008bfb5e/e1daffb4-2a86-4233-b0c7-02d26b3da171.pdf.

[13] Office of the Ministry of Education. Notice of the General Office of the Ministry of Education on carrying out action to improve the ability of vocational education teachers. 2022. http://www.moe.gov.cn/srcsite/A10/s7034/202205/t20220523_629603.html

[14] OECD. OECD skills outlook 2019. Organisation for Economic and Co-operation and Development. 2019. 10.1787/df80bc12-en.

[15] Alamutka K. Mapping digital competence: towards a conceptual understanding. Institute for Prospective Technological Studies. 2011. https://www.semanticscholar.org/paper/Mapping-Digital-Competence:-Towards-a-Conceptual-Ala-Mutka/dd8bb2ae8ae95b9b91c3d623581f3b4a08c5bbb5.

[16] Calvani A., Cartelli A., Fini A., & Ranieri M. Models and instruments for assessing digital competence at school. Journal of e-Learning and Knowledge Society, 2008. 4(3), pp.183–193. https://www.researchgate.net/publication/288948663_Models_and_Instruments_for_assessing_Digital_Competence_at_School.

[17] Commissie E. Digital competence in practice: an analysis of frameworks. 2012. https://op.europa.eu/en/publication-detail/-/publication/2547ebf4-bd21-46e8-88e9-f53c1b3b927f/language-en.

[18] Xiao J. Digital literacy. Chinese Journal of Distance Education, 2006 (05): 32–33. https://kns.cnki.net/kcms2/article/abstract?v=3uoqIhG8C44YLTlOAiTRKgchrJ08w1e7eWoVfj7plMw90iQKWQD2KwtH0_25ejozo8KvikuQK3ifczLkLkc1U1flvxN3t0fO&uniplatform=NZKPT.

[19] Ren Y., Yang X. Digital Competency: an indispensable capability in the information age. Digital Teaching in Primary and Secondary Schools, 2017 (01): 22–24. https://kns.cnki.net/kcms2/article/abstract?v=3uoqIhG8C44YLTlOAiTRKibYlV5Vjs7iNJsczffWK8Ot9R_qK5HGp0MiBWqlncd_pL36f1paAJrMHCRfY3bTjfqcf0q_g0Kw&uniplatform=NZKPT.

[20] Office of the Central Cyberspace Affairs Commission. Platform for Action to enhance Digital literacy and skills for all. 2021. http://www.cac.gov.cn/2021-11/05/c_1637708867754305.htm.

[21] Zheng X., Fan X. Research on Digital Competence of EU Citizens: A Comparative Analysis Based on the Dig Comp1.0, 2.0 and 2.1. International and Comparative Education, 2020, 42 (06): 26–34. https://kns.cnki.net/kcms2/article/abstract?v=3uoqIhG8C44YLTlOAiTRKibYlV5Vjs7i8oRR1PAr7RxjuAJk4dHXos4axk50dH0J-JO_e4FLABEE-2LeTVe3DjpVm2PaDrSa&uniplatform=NZKPT.

[22] Redecker C. European Framework for the Digital Competence of Educators: DigCompEdu. 2017. https://joint-research-centre.ec.europa.eu/digcompedu_en#:~:text=The%20European%20Framework%20for%20the%20Digital%20Competence%20of,the%20development%20of%20educator-specific%20digital%20competences%20in%20Europe.

[23] ISTE. ISTE standards for educators. 2017. https://beta.iste.org/standards/educators#:~:text=ISTE%20Standards%3A%20For%20Educators%201%202.1%20Learner%20Educators,…%206%202.6%20Facilitator%20…%207%202.7%20Analyst.

[24] Kelentri´c M.; Helland K.; Arstorp A.T. Professional Digital Competence Framework for Teachers. The Norwegian Centre for ICT in Education: Oslo, Norway. 2017. https://www.udir.no/globalassets/filer/in-english/pfdk_framework_en_low2.pdf#:~:text=The%20Professional%20Digital%20Competence%20Framework%20for%20Teachers%20is,education%20and%20systematic%20continuing%20professional%20development%20of%20teachers.

[25] JISC. Digital Teaching Professional Framework. 2023. https://www.et-foundation.co.uk/professional-developm-ent/edtech-support/digital-skills-competency-framework/.

[26] INTEF. Common Digital Competence Framework for Teachers. 2017. https://aprende.intef.es/sites/default/files/2018-05/2017_1024-Common-Digital-Competence-Framework-For-Teachers.pdf.

[27] Pérez K. V. P., Torelló O. M. The digital competence as a cross-cutting axis of higher education teachers’ pedagogical competences in the European higher education area. Procedia-Social and Behavioral Sciences. 2012.46: 1112–1116. https://www.sciencedirect.com/science/article/pii/S1877042812013869.

[28] Morellato M. Digital competence in tourism education: Cooperative-experiential learning. Journal of Teaching in Travel & Tourism. 2014.14(2):184–209. https://www.tandfonline.com/doi/abs/10.1080/15313220.2014.907959.

[29] Krumsvik R. J., Jones L.Ø., Øfstegaard M., Eikeland O.J. Upper secondary school teachers’ digital competence: analysed by demographic, personal and professional characteristics. Nordic Journal of Digital Literacy. 2016.10(3):143–164. https://www.researchgate.net/publication/309225756_Upper_Secondary_School_Teachers’_Digital_Competence_Analysed_by_Demographic_Personal_and_Professional_Characteristics.

[30] Instefjord E., Munthe E. Preparing pre-service teachers to integrate technology: an analysis of the emphasis on digital competence in teacher education curricula. European Journal of Teacher Education. 2016.39(1):77–93. https://www.tandfonline.com/doi/abs/ doi: 10.1080/02619768.2015.1100602

[31] Zheng X. Research on the Construction and Application of Digital Competence Model for K12 Teachers in China. East China Normal University, 2019. https://kns.cnki.net/kcms2/article/abstract?v=3uoqIhG8C447WN1SO36whLpCgh0R0Z-iDdIt-WSAdV5IJ_Uy2HKRAcEhNOhKb2Y8Tzv4rZxY9FLz5Qn4wZurDxcqOXs-wt5e&uniplatform=NZKPT.

[32] Sun X., Li Q. International Experience of "Learner-centered" Framework of Digital Competence for Teachers. Journal of Comparative Education. 2022.(01):28–40. https://kns.cnki.net/kcms2/article/abstract?v=3uoqIhG8C44YLTlOAiTRKibYlV5Vjs7iJTKGjg9uTdeTsOI_ra5_XTr0p5tQza6NZJE7V6c29vwApmAI1q2wbEPTzAE0cVXX&uniplatform=NZKPT.

[33] Ministry of Education of the People’s Republic of China. Notice of the Ministry of Education on the release of "Digital Campus Standards for Vocational Colleges". 2020. http://www.moe.gov.cn/srcsite/A07/zcs_zhgg/202007/t20200702_469886.html

[34] Wang Z.; Chu Z. Examination of Higher Education Teachers’ Self-Perception of Digital Competence, Self-Efficacy, and Facilitating Conditions: An Empirical Study in the Context of China. Sustainability 2023, 15, 10945. 10.3390/su151410945.

[35] Almerich G., Orellana N., Suárez-Rodríguez J., & Díaz-García I. Teachers’ information and communication technology competences: A structural approach. Computers & Education. 2016.100, 110–125. 10.1016/j.compedu.2016.05.002.

[36] Sipila K. Educational use of information and communications technology: Teachers’ perspective. Technology, Pedagogy and Education. 2014 23(2), 225–241. 10.1080/1475939X.2013.813407.

[37] Krumsvik R. J., Jones L.Ø., Øfstegaard M., & Eikeland O. J. Upper secondary school teachers’ digital competence: Analysed by demographic, personal and professional characteristics. Nordic Journal of Digital Literacy. 2016. 3(11), 143–164. 10.18261/issn.1891-943x-2016-03-02.

[38] Fraile M. N., Penalva-V ˜ ´elez A., & Lacambra A. M. M. Development of digital competence in secondary education teachers’ training. Education Sciences. 2018.8 (104), 1–12. 10.3390/educsci8030104.

[39] Tondeur J., Aesaert K., Prestridge S., & Consuegra E. (2018). A multilevel analysis of what matters in the training of pre-service teacher’s ICT competencies. Computers & Education. 2018.122, 32–42. doi: 10.1016/j.compedu.2018.03.002

[40] Ryan R. M., & Deci E. L. (2017). Self-determination theory: Basic psychological needs in motivation development and wellness. Guilford Press.

[41] Chiu T. K., Luke B., Chai C. S., & Ismailov M. (2023). Teacher support and student motivation to learn with Artificial Intelligence (AI) based chatbot. Interactive Learning Environments, 1–17. 10.1080/10494820.2023.2172044.

[42] Qin F., & Yan J. (2020). Understanding user trust in artificial intelligence-based educational systems: Evidence from China. British Journal of Educational Technology, 51(5), 1693–1710. 10.1111/bjet.12994.

[43] Xia Q., Chiu T. K. F., Lee M., Temitayo I., Dai Y., & Chai C. S. (2022). A self-determination theory (SDT) design approach for inclusive and diverse artificial intelligence (AI) education. Computers & Education, 189, 158–162. 10.1016/j.compedu.2022.104582.

[44] Hinojo-Lucena F., Aznar-Díaz I., Caceres-Reche M., Trujillo-Torres J., & Romero-Rodríguez J. (2019). Factors influencing the development of digital competence in teachers: Analysis of the teaching staff of permanent education centres. IEEE Access, 7, 178744–178752. 10.1109/ACCESS.2019.2957438.

[45] Ghomi, M., & Redecker, C. (2019). Digital competence of educators (DigCompEdu): Development and evaluation of a self-assessment instrument for teachers’ digital competence. Proceedings of the 11th International Conference on Computer Supported Education (CSDU 2019), 1, 541–548.

[46] INTEF (2022). Competencia digital docente. https://intef.es/competencia-digital-educativa/competencia-digital-docente/.

[47] Ochoa Juan & Claro Magdalena & Hinostroza J. & Castro-Grau Carolina & Cabello Patricio. (2024). Systematic review of quantitative research on digital competences of in-service school teachers. Computers & Education. doi: 10.1016/j.compedu.2024.105030

[48] Andronic Adrian. (2024). Dual Education Digitalization: Unpacking Financial Strategies across Europe. 275–289. doi: 10.53486/dri2023.20

[49] Andronic Adrian. (2024). Dual Education Digitalization: Unpacking Financial Strategies across Europe. 275–289. doi: 10.53486/dri2023.20

[50] Dissertation & O’Gara, Dorothy & California,. (2023). Mobile Technology Integration, Teacher Self-efficacy, and Student Achievement in New Hampshire: A Correlational Study.

[51] Birgin O., Uzun K., & Mazman Akar S. G. (2020). Investigation of Turkish mathematics teachers’ proficiency perceptions in using information and communication technologies in teaching. Education and Information Technologies, 25(1), 487–507. doi: 10.1007/s10639-019-09977-1

[52] Ochoa Juan & Claro Magdalena & Hinostroza J. & Castro-Grau Carolina & Cabello Patricio. (2024). Systematic review of quantitative research on digital competences of in-service school teachers. Computers & Education. doi: 10.1016/j.compedu.2024.105030

[53] Saputro N. E., Purnomo P., Tuwoso T. Knowledge, mechanical aptitude, and age on dual-expertise teacher competence: a correlational study. Jurnal Pendidikan Sains Universitas Negeri Malang. 2019.vol. 7, no. 4, 30 (4). https://www.neliti.com/publications/478741/knowledge-mechanical-aptitude-and-age-on-dual-expertise-teacher-competence-a-cor#cite.

[54] Yonggang Feng, Ying Chen. The Loss and Reshaping of Teacher Moral Authority in the Digital Media Era. Contemporary Education Science, 2021, (05): 31–38.

[55] Yi Li, Sirui Wu, Qin Liao. Research on the influencing factors and moderating effects of teachers’ use of information technology: based on the UTAUT model. China Electronics Education, 2016, (10): 31–38.

[56] Xu Fang, Xinhua Zhang, Lin Li. Factors influencing teachers’ behavioral intentions on STEM online education platforms: a survey based on the Wise platform of South China Normal University. Open Education Research, 2018, 24 (03): 59–67. doi: 10.13966/j.cnki.kfjyyj.2018.03.007,

[57] Xuan Wang, Tingting Gao, Jun Tian, et al. Strong interactive delivery classroom design and teacher-student acceptance analysis. China Electronic Education, 2021, (12): 95–102+138.

[58] Zhiyong Zheng, Qingze Fan, Wei Jia. The Triple Illusion of Empowering Teacher Development with Artificial Intelligence Technology and the Solution. China Electronic Education, 2024, (07): 28–34+73.

[59] Xudong Zheng, Yunfei Ma, Tingyan Yue. Continuously promoting the professional development of teachers in the digital age: an investigation based on the Norwegian Teacher Professional Digital Competence Framework. Journal of Comparative Education, 2021, (01): 139–150.

[60] Romero-Hall E, Jaramillo Cherrez N (2022) Teaching in times of disruption: faculty digital literacy in higher education during the COVID-19 pandemic. Innovations in Education and Teaching International 1–11. 10.1080/14703297.2022.2030782.

[61] Halverson R., & Shapiro R. B. (2012). Technologies for education and technologies for learners: How information technologies are (and should be) changing schools. Wisconsin Center for Educational Research (WCER), Working Paper, 6.

[62] NT. Fenwick, R. Edwards, Exploring the impact of digital technologies on professional responsibilities and education, European Educational Research Journal 15 (1) (2016) 117–131.

[63] Williamson B., Eynon R., Potter J. Pandemic politics, pedagogies and practices: digital technologies and distance education during the coronavirus emergency. Learning, Media and Technology, 45 (2) (2020), pp. 107–114.

[64] LaMonica H.M., Davenport T.A., Roberts A.E., Hickie I.B. Understanding technology preferences and requirements for health information technologies designed to improve and maintain the mental health and well-being of older adults: Participatory design study. JMIR aging, 4 (1) (2021), p. e21461. doi: 10.2196/21461 33404509 PMC7817357

[65] Camilleri M.A., Camilleri A.C. The acceptance of learning management systems and video conferencing technologies: Lessons learned from COVID-19 Technology, Knowledge and Learning (2021), pp. 1–23

[66] OECD (2023), "Monitoring and evaluation of digital education", in Shaping Digital Education: Enabling Factors for Quality, Equity and Efficiency, OECD Publishing, Paris, 10.1787/38f932e1-en.

[67] Lan Thi Nguyen Issara Kanjug, Lowatcharin Grichawat, Manakul Theeradej, Poonpon Kornwipa, Sarakorn Weerachai, Somabut Anucha, Srisawasdi Niwat, Traiyarach Saksuriya, Tuamsuk Kulthida, How teachers manage their classroom in the digital learning environment–experiences from the University Smart Learning Project, Heliyon, Volume 8, Issue 10, 2022, e10817, ISSN 2405-8440, 10.1016/j.heliyon.2022.e10817 PMC9547237 36217475

[68] Gottschalk F. and Weise C. (2023), "Digital equity and inclusion in education: An overview of practice and policy in OECD countries", OECD Education Working Papers, No. 299, OECD Publishing, Paris, 10.1787/7cb15030-en.

[69] Pinho C., Franco M., & Mendes L. (2021). Application of innovation diffusion theory to the E-learning process: higher education context. Education and Information Technologies, 26(1), 421–440.

[70] Tokareva E.A., Smirnova Y.V., Orchakova L.G. Innovation and communication technologies: Analysis of the effectiveness of their use and implementation in higher education. Education and Information Technologies, 24 (5) (2019), pp. 3219–3234.

[71] Goriss-Hunter A., Sellings P. & Echter A. Information Communication Technology in schools: Students Exercise ‘Digital Agency’ to Engage with Learning. Tech Know Learn 27, 785–800 (2022). 10.1007/s10758-021-09509-2.

[72] OECD (2023), "Monitoring and evaluation of digital education", in Shaping Digital Education: Enabling Factors for Quality, Equity and Efficiency, OECD Publishing, Paris, 10.1787/38f932e1-en.

[73] Cabero-Almenara J., Guillén-Gámez F.D., Ruiz-Palmero J., Palacios-Rodríguez A. Digital competence of higher education professor according to DigCompEdu. Statistical research methods with ANOVA between fields of knowledge in different age ranges. Education and Information Technologies (2021), pp. 1–18. 10.1007/s10639-021-10476-5 PMC7971399 33758572

[74] Wu M. SPSS statistical application practice: questionnaire analysis and applied statistics. Science Press.2003. https://book.sciencereading.cn/shop/book/Booksimple/onlineRead.do?id=BBD30E06AD52D4BC5BEC13EFE3994F429000&readMark=0.

[75] Sang P. Li J. Risk Evaluation of Prefabricated Buildings Development and Construction Based on Structural Equation. Journal of Civil Engineering and Management.2017.34(04):89–95. https://kns.cnki.net/kcms2/article/abstract?v=3uoqIhG8C44YLTlOAiTRKibYlV5Vjs7iAEhECQAQ9aTiC5BjCgn0RgqxLNOEDLHvLKECz1d6CT1wFB1ss6Ges6X1-B_qCoZJ&uniplatform=NZKPT.

[76] Yan H., Li X., Ren Y. Development and Validation of Self-measurement Tools for Pre-service Teachers’ ICT Competency. e-Education Research, 2018,39(01):98–106. https://kns.cnki.net/kcms2/article/abstract?v=3uoqIhG8C44YLTlOAiTRKibYlV5Vjs7i0-kJR0HYBJ80QN9L51zrP9OeLGwb8OABVv0TFukEzCO1AKsg8xWHt36ZNT05ANP7&uniplatform=NZKPT.

[77] Long C., Song X., Wang A. Analysis on Regional Disequilibrium and Convergence of China’s Economic Development. Statistics & Decision. 2023,39(07):106–112. https://kns.cnki.net/kcms2/article/abstract?v=3uoqIhG8C44YLTlOAiTRKibYlV5Vjs7ioT0BO4yQ4m_mOgeS2ml3UG7pGpZzhP1YIen4ypbxBqDr0HNtycK_IfqtWU_ZhklR&uniplatform=NZKPT.

[78] Gao X., Wei F., Zhou X. An Ecosystem Perspective on Why Teachers Burnout: Analysis based on Ecosystem Theory. Research in Educational Development. 2023,43(02):44–51. https://kns.cnki.net/kcms2/article/abstract?v=3uoqIhG8C44YLTlOAiTRKibYlV5Vjs7ioT0BO4yQ4m_mOgeS2ml3UJ5KOlzi3Wr_zN8RPCL9TLyD7Fgaba6v5MZWd7Z0H3Ub&uniplatform=NZKPT.

[79] Shao G., Wei Q., Zhang Y. Research on Teachers’ inertia in the perspective of educational reform. Teacher Education Research.2020,32(05):76–82. https://kns.cnki.net/kcms2/article/abstract?v=3uoqIhG8C44YLTlOAiTRKibYlV5Vjs7i8oRR1PAr7RxjuAJk4dHXoo8kmqh3pH8J166tz9u41o0JgLG8a5dV3BEiCfahqLro&uniplatform=NZKPT.

[80] Jennifer A. K., Mindy K. B. The evaluation and impact of educational media and technology on children and adolescents, Encyclopedia of Child and Adolescent Health. Academic Press, 2023, Pages 408–416,I SBN 9780128188736. 10.1016/B978-0-12-818872-9.00174-6

[81] Du L. Higher Vocational Education and Regional Economy. Learning & Education. 2020. 9. 20. https://www.researchgate.net/publication/348267639_Higher_Vocational_Education_and_Regional_Economy

[82] Julian F., John A., Wolfram S., Tim F., Eveline G. Preparing for life in a digital age: The IEA international computer and information literacy study international report. Springer. 2014. https://link.springer.com/content/pdf/10.1007/978-3-319-14222-7.pdf?pdf=button.

[83] Claro M., Salinas A., Cabello-Hutt T., San Martín E., Preiss D. D., Valenzuela S., et al. Teaching in a digital environment (TIDE): Defining and measuring teachers’ capacity to develop students’ digital information and communication skills. Computers & Education, 2018.121, 162–174. 10.1016/j. compedu.2018.03.001.

[84] OECD. TALIS 2018 results (volume I): Teachers and school leaders as lifelong learners. Paris, France: OECD Publishing. 2019. 10.1787/1d0bc92a-en.

[85] OECD. Curriculum flexibility and autonomy in Portugal–an OECD review. Paris, France: Directorate for Education and Skills–OECD. 2018. https://www.oecd.org/education/2030/Curriculum-Flexibility-and-Autonomy-in-Portugal-an-OECD-Review.pdf.

